# Compounds Isolated from *Paepalanthus* spp. (Eriocaulaceae) and Their Evaluation in Antimicrobial, Cytotoxic, and Antiviral Assays

**DOI:** 10.1021/acsomega.4c11026

**Published:** 2025-06-18

**Authors:** Laysa Lanes Pereira Ferreira Moreira, Lucas Almeida Oliveira, Raphael Conti, Larissa Costa de Almeida, Leticia V. Costa-Lotufo, Ana Camila Micheletti, Isabela Dolci, Rafaela Sachetto Fernandes, Glaucius Oliva, Rafael Victorio Carvalho Guido, Valdemar Lacerda, Keyller Bastos Borges, Warley de Souza Borges

**Affiliations:** † Departamento de Química, 28126 Universidade Federal Espírito Santo, Avenida Fernando Ferrari 514, Goiabeiras, Vitória, Espírito Santo 29075-910, Brazil; ‡ Departamento de Farmacologia, 28133 Universidade de São Paulo, Avenue Prof. Lineu Prestes, 1524, Butantã,São Paulo, São Paulo 05508-000, Brazil; § Instituto de Química, 54534 Universidade Federal do Mato Grosso do Sul, Avenida Senador Filinto Müller, 1555, Cidade Universitária, Campo Grande, Mato Grosso do Sul 79074-460, Brazil; ∥ Instituto de Física de São Carlos, 28133 Universidade de São Paulo, Avenue João Dagnone, 1100, Jardim Santa Angelina, São Carlos, São Paulo 13563-120, Brazil; ⊥ Departamento de Ciências Naturais, Universidade Federal de São João del-Rei, Campus Dom Bosco, Praça Dom Helvécio 74, Fábricas, São João del-Rei, Minas Gerais 36301-160, Brazil

## Abstract

*Paepalanthus acanthophyllus* and *P. bromelioides* belong to the Eriocaulaceae family. According to the literature, secondary metabolites isolated in *Paepalanthus* have interesting biological activities. This research aimed to isolate constituents from the capitula of the mentioned species and to evaluate their cytotoxic, antimicrobial, and antiviral activities. Through maceration and several types of separation procedures, the crude extracts were produced, and the structures of constituents were identified mostly by nuclear magnetic resonance. Cytotoxic and antimicrobial activities were evaluated by 3-(4,5-dimethylthiazol-2-yl)-2,5-diphenyltetrazolium bromide and 2,3,5-triphenyl-2*H*-tetrazolium chloride methods, respectively, while antiviral activity was evaluated by enzymatic and phenotypic assays. Two flavonoids were isolated in the dichloromethane fraction, and four flavonoids and one benzoic acid derivative were isolated in the ethyl acetate fraction of *P. acanthophyllus*. Paepalantine was isolated from *P. bromelioides*. The isolated compounds did not show significant cytotoxicity, and the highest values of growth-inhibitory activity observed reached 29.17% (colon cancer) and 19.24% (breast cancer). Antimicrobial evaluation showed the best result to 6-methoxykaempferol against *S. aureus* (MIC = 250 μg mL^–1^). Antiviral evaluation showed that 6-methoxykaempferol-3-*O*-β-D-6″-(*p*-coumaroyl)-glucopyranoside inhibited the ZIKV enzyme NS2B-NS3pro in the lowest micromolar range (IC_50_ = 1.95 μM); however, it did not show inhibition when evaluated in phenotypic assays with the ZIKV replicon.

## Graphical abstract

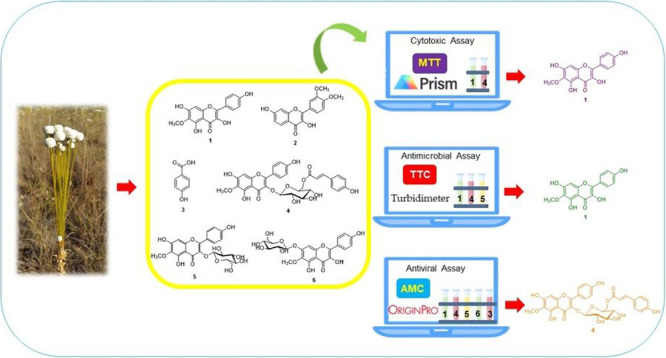

## Introduction

Eriocaulaceae is a pantropical family, known for morphologically presenting scapes and capitula inflorescences. It has its greatest variety of species in Brazil and Venezuela, where the highest rate of endemism can also be found.^1,2^ In Brazil, Eriocaulaceae presents 605 species, distributed in eight genera,^3,4^ with *Eriocaulon*, *Paepalanthus,* and *Syngonanthus* being the most chemically studied genera.

*Paepalanthus* is found in the African and American continents, with its largest expression being in the American continent. In general, the genus is composed of approximately 410 species. In Brazil, there are 341 species, 327 of which are endemic, while *Paepalanthus* species can be found all over the country, and the largest diversity is found at “Cadeia do Espinhaço”, located in Minas Gerais and Bahia states, where it is estimated that approximately 200 species occur with 82% of the endemic species.^3,5,6^

Among *Paepalanthus* species, there are *Paepalanthus acanthophyllus* and *Paepalanthus bromelioides*, and both species are endemic to Brazil. *P. acanthophyllus* is found in Goiás State,^7,8^ and according to the literature, there is only one study on *P. acanthophyllus* constituents, which describes the isolation of the flavonoids: 6-methoxykaempferol-3-*O*-(6″-*p*-coumaroyl)-β-d-glucopyranosyl-7-*O*-β-d-glucopyranoside, 6-methoxykaempferol-3-7-di-*O*-β-d-glucopyranoside, 6-methoxykaempferol-3-*O*-β-d-glucopyranoside, and naphthopyranones paepalantine-9-*O*-β-d-glucopyranoside and paepalantine.^7^ These first two flavonoids were reported for the first time in the genus. Additionally, the antimicrobial and cytotoxic activities of the methanol extract of this species have been evaluated.^7^ For the evaluation of the antimicrobial activity, Gram-positive and Gram-negative bacterial strains were used, and values greater than 1000 μg·mL^–1^ were observed for all strains analyzed ( *Escherichia coli* ATCC 25922, *Staphylococcus aureus* ATCC 25923, and *Salmonella setubal* ATCC 19196). The methanol extract was inactive, as well.^9^ For the evaluation of the cytotoxic activity, the 3-(4,5-dimethylthiazol-2-yl)-2,5-diphenyltetrazolium bromide (MTT) method was used, and an IC_50_ value equal to 34.81 μg mL^–1^ was obtained for the MCF-7 lineage. These results suggested that the components of the methanolic extract showed promising inhibitory activity of the human breast cancer cells.^7^ *P. bromelioides* is found in the state of Minas Gerais. It is commonly known as “sempre-viva” and “capipoatinga”.^10,11^ Unlike *P. acanthophyllus*, the species *P. bromelioides* have been submitted to several chemical and biological studies, including the isolation of naphthopyranone paepalantine.^12–14^

Several biological activities were reported on substances from flavonoid and naphthopyranone classes.^14–18^ For instance, flavonoids have been showing positive results for antitumor, antimicrobial, and antiviral activities.^19–30^

Many researchers have sought to understand the relationship between the chemical structures of compounds and their biological activity. When it comes to flavonoids, the presence of the double bond between the C2=C3 carbons in conjunction with the oxo group on the C4=O carbon is the main contributing factor for these activities.^31^ Moreover, there are other biological factors and contributions, mostly related to cancer research.^32–36^

Thus, the aim of the present work was to carry out a phytochemical investigation of *P. acanthophyllys* and *P. bromelioides* capitula to isolate bioactive compounds using classical and modern isolation techniques. The structures of isolated constituents were identified mostly by nuclear magnetic resonance. Furthermore, an assessment was carried out to evaluate antitumor, antimicrobial, and antiviral activities of compounds. Antitumor and antimicrobial activities were performed using known and well-established methods such as MTT and TTC, respectively. Antiviral activity was evaluated by enzymatic and phenotypic assays.

## Results and Discussion

### Elucidation of Isolated Compounds

Six phenolic compounds were isolated from the species *P. acanthophyllus* and one compound from the species *P. bromelioides* (PbD-01). For structural identification of the compounds, ^1^H and ^13^C NMR and two-dimensional COSY, HSQC, and HMBC analyses were performed. The chemical shifts were compared to those in the literature, which corroborated the structural determination. It should be noted that compounds **1** and **4** are new to the species but have already been isolated in the genus. Compounds **2**, **3,** and **6** are new to the Eriocaulaceae family.^37–46^ Compound **5** and PbD-01 were previously isolated in the corresponding species. In this section, since these are compounds already known in the literature, only chemical shifts are presented. Figure 1 shows the structure of the compounds isolated in *P. acanthophyllus* and the two-dimensional couplings. Figure 2 shows the structure of compound PbD-01 and its chemical shifts. Two-dimensional analyses of PbD-01 were not performed because it is a compound frequently isolated in *P. bromelioides*.

**Figure 1:**
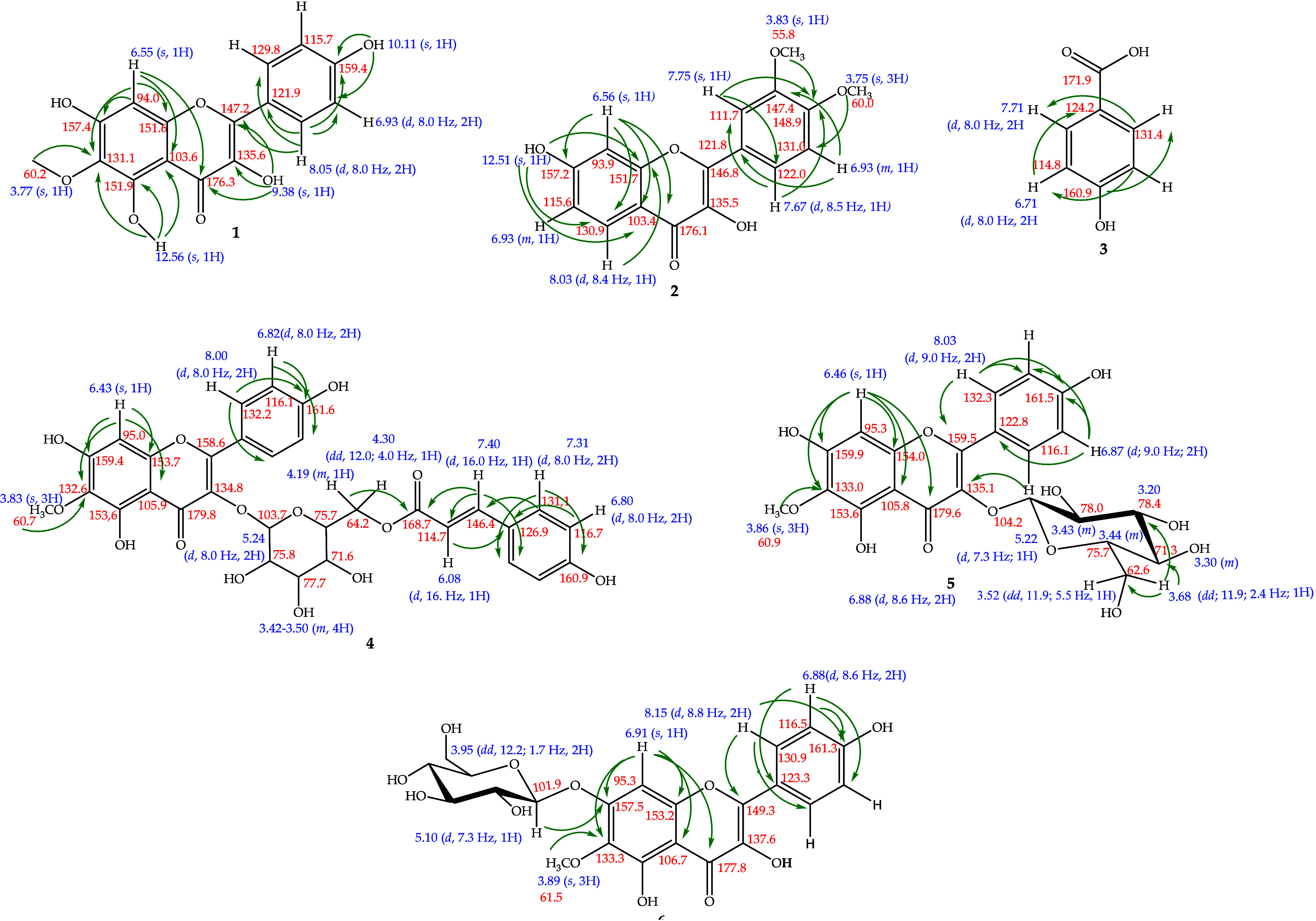
Chemical constituents isolated from the capitula of *P. acanthophyllus*.

**Figure 2:**
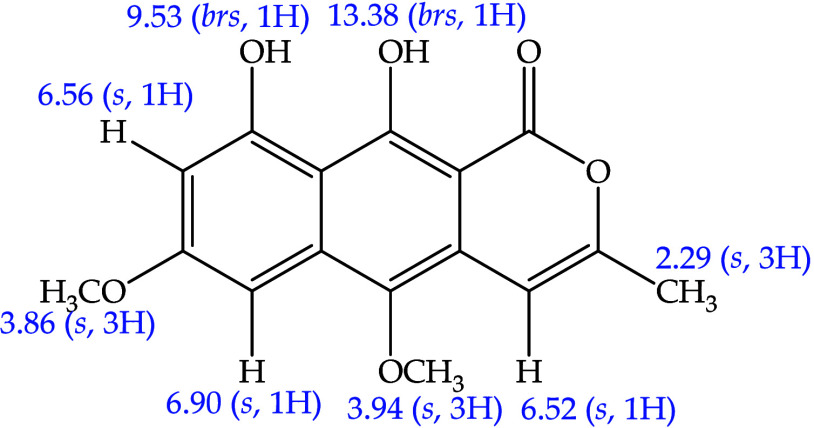
Paepalantine structure; compound isolated from the capitula of *P. bromelioides*.

#### Paepalanthus Acanthophyllus

##### Compound **1**: 6-Methoxykaempferol^37^

^1^H NMR (DMSO-*d*_6_, 400 MHz): δ_H_ 6.55 (1H, s, H8), 6.93 (2H, d, *J* = 8.0 Hz, H3′ and H5′), 8.05 (2H, d, *J* = 8.0 Hz, H2′ and H6′), 3.77 (3H, s, 3-OCH_3_). ^13^C NMR (DMSO-*d*_6_, 100 MHz): δ_C_ 147.2 (C2), 135.6 (C3), 176.3 (C4), 151.9 (C5), 131.1 (C6), 157.4 (C7), 94.0 (C8), 151.6 (C9), and 103.6 (C10), 121.9 (C1’), 129.8 (C2’ and C6’), 115.7 (C3′ and C5′), 159.4 (C4′), 60.2 (6-OCH_3_).

##### Compound **2**: 3′,4′-Dimethoxyfisetin^38^

^1^H NMR (DMSO-*d*_6_, 400 MHz): δ_H_ 8.03 (1H, d, *J* = 8.4 Hz, H5), 6.93 (2H, m, H6 and H5′), 6.56 (1H, s, H8), 7.75 (1H, s, H2’), 7.67 (1H, d, *J* = 8.5 Hz, H6’), 3.83 (3H, s, 3′-OCH3), 3.75 (3H, s, 4’-OCH_3_), 9.64 (1H, s, 3-OH), 12.53 (1H, s, 7-OH). ^13^C NMR (DMSO-*d*_6_, 100 MHz): δ_C_ 146.8 (C2), 135.5 (C3), 176.1 (C4), 130.9 (C5), 115.6 (C6), 157.2 (C7), 93.9 (C8), 151.7 (C9), 103.4 (C10), 121.8 (C1’), 111.8 (C2’ and C6’), 147.4 (C3′ and C5′), 148.9 (C4’), 55.8 (3′-OCH_3_), 60.0 (4’-OCH_3_).

##### Compound **3**: *p*-Hydroxybenzoic Acid^39,40^

^1^H NMR (DMSO-*d*_6_, 400 MHz): δ_H_ 6.71 (2H, d, *J* = 8.0 Hz, H3 and H5), δ_H_ 7.71 (2H, d, *J* = 8.0 Hz, H2 and H6). ^13^C NMR (DMSO-*d*_6_, 100 MHz): δ_C_ 124.2 (C1), 131.4 (C2 and C6), 114.8 (C3 and C5), 160.9 (C4).

##### Compound **4**: 6-Methoxykaempferol-3-*O*-β-D-6″-(*p*-coumaroyl)-glycopyranoside^41,42^

^1^H NMR (CD_3_OD, 400 MHz): δ_H_ 6.43 (1H, s, H8), 8.00 (2H, d, *J* = 8.0 Hz, H2′ and H6′), 6.82 (2H, d, *J* = 8.0 Hz, H3′ and H5′), 3.83 (3H, s, 6-OCH_3_), 5.24 (1H, d, *J* = 8.0 Hz, H1″), 3.42–3.50 (4H, m, H2″, H3″, H4″, and H5”), 4.30 (1H, dd, *J* = 12.0, 4.0 Hz, H6a″), 4.19 (1H, m, H6b″), 7.31 (2H, d, *J* = 8.0, H2″′ and H6″′), 6.80 (2H, d, *J* = 8.0, H3″′ and H5″′), 6.08 (2H, d, *J* = 16.0, Ha), 7.40 (2H, d, *J* = 16.0, Hb). ^13^C NMR (CD_3_OD, 100 MHz): δ_C_ 158.6 (C2), 134.8 (C3), 179.8 (C4), 153.6 (C5), 132.6 (C6), 159.4 (C7), 95.0 (C8), 153.7 (C9), 105.9 (C10), 122.7 (C1′), 132.2 (C2′ and C6′), 116.1 (C3′ and C5′), 161.6 (C4′), 60.7 (6-OCH_3_), 103.7 (C1″), 75.8 (C2″), 77.7 (C3″), 71.6 (C4″), 75.7 (C5″), 64.2 (C6a′), 64.2 (C6b′), 126.9 (C1″′), 131.1 (C2″′ and C6″′′), 116.7 (C3″′ and C5″′), 160.0 (C4″′), 146.4 (C7″′), 114.7 (C8″′), 168.7 (C9′).

##### Compound **5**: 6-Methoxykaempferol-3-*O*-β-glucopyranoside^7^

^1^H NMR (CD_3_OD, 400 MHz): δ_H_ 6.46 (1H, s, H8), 8.03 (2H, d, *J* = 9.0 Hz, H2′ and H6′), 6.87 (2H, d, *J* = 9.0 Hz, H3′ and H5′), 3.86 (3H, s, 6-OCH_3_), 5.22 (1H, d, *J* = 7.3 Hz, H1″), 3.44–3.20 (4H, m, H2″, H3″, H4″, and H5″), 3.68 (1H, dd, *J* = 11.9, 2.4 Hz, H6a″), 3.52 (1H, dd, *J* = 11.9, 5.5 Hz, H6b″). ^13^C NMR (CD_3_OD, 100 MHz): 159.5 (C2), 135.1 (C3), 179.6 (C4), 153.6 (C5), 133.0 (C6), 159.9 (C7), 95.3 (C8), 154.0 (C9), 105.8 (C10), 122.8 (C1′), 132.3 (C2′ and C6′), 116.1 (C3′ and C5′), 161.5 (C4′), 60.0 (3′-OCH_3_), 104.2 (C1″), 75.7 (C2″), 78.0 (C3″), 71.3 (C4″), 78.4 (C5″), 62.6 (C6″).

##### Compound **6**: 6-Methoxykaempferol-7-*O*-β-d-glucopyranoside^7^

^1^H NMR (CD_3_OD, 400 MHz): δ_H_ 6.91 (1H, s, H8), 8.15 (2H, d, *J* = 8.8 Hz, H2′ and H6′), 6.88 (2H, d, *J* = 8.6 Hz, H3′ and H5′), 3.89 (3H, s, 6-OCH_3_), 5.10 (1H, d, *J* = 7.3 Hz, H1″), 3.75–3.40 (4H, m, H2″, H3″, H4″, and H5″), 3.96 (1H, dd, *J* = 12.2, 1.7 Hz, H6a″ and H6b″). ^13^C NMR (CD_3_OD, 100 MHz): 149.3 (C2), 137.6 (C3), 177.8 (C4), 153.1 (C5), 133.3 (C6), 157.5 (C7), 95.3 (C8), 153.2 (C9), 106.7 (C10), 123.3 (C1′), 130.9 (C2′ and C6′), 116.5 (C3′ and C5′), 161.3 (C4′), 61.5 (3′-OCH_3_), 101.9 (C1″), 77.9 (C2″), 78.5 (C3″), 71.3 (C4″), 74.8 (C5″), 62.5 (C6″).

#### Paepalanthus Bromelioides

##### Compound PbD-01: Paepalantine^7,14^

^1^H NMR (CD_3_OD, 400 MHz): δ_H_ 6.52 (1H, s, H4), 6.90 (1H, s, H6), 6.56 (1H, s, H8), 3.94 (3H, s, 5-OCH_3_), 3.86 (3H, s, 7-OCH_3_), 9.53 (1H, br, 9-OH), 13.38 (1H, br, 10-OH), 2.29 (3H, s, 11-CH_3_).^7,14^

### Evaluation of the Biological Activities

#### Cytotoxic Activity Evaluation

The growth-inhibitory activities of isolated compounds **1** and **4** were tested by an in vitro MTT assay at the concentration of 50 μM using two human cancer cell lines HCT-116 (human colon cancer) and MCF-7 (human breast cancer).

In this study, high cytotoxic activity was considered for compounds that showed an inhibition of cell growth greater than 70%. As shown in Figure 3, the tested compounds did not show high cytotoxicity against both the HCT-116 and MCF-7 cells at a concentration of 50 μM. Doxorubicin, as expected, inhibited both cell lines with half-maximal inhibition concentration (IC_50_) values of 0.18 μM (0.13–0.23 μM) in HCT-116 and 1.16 μM (0.86–1.55 μM) in MCF-7.

**Figure 3:**
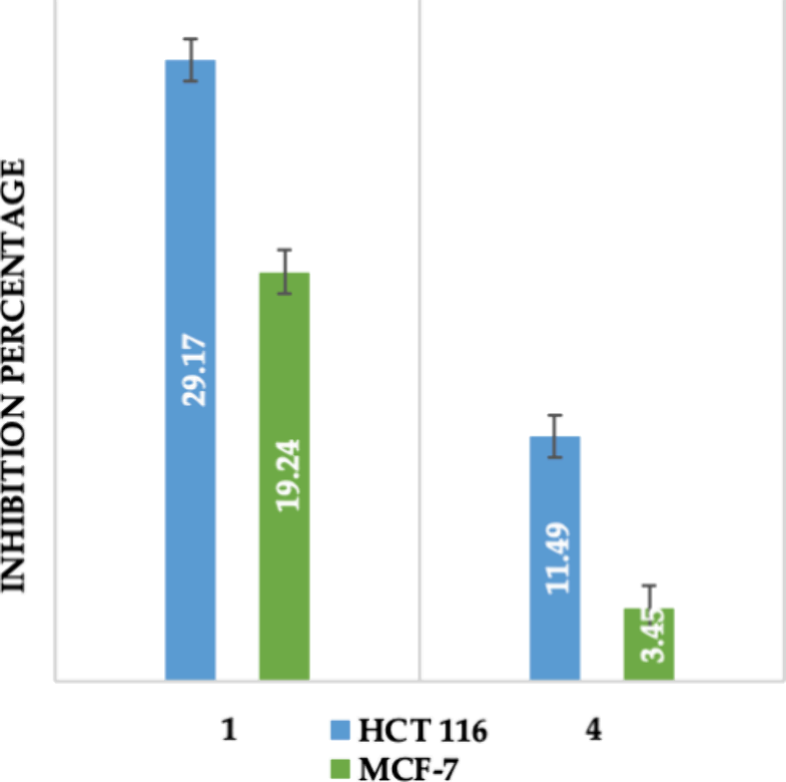
Percentage of inhibitory growth of isolated constituents **1** and **4** in *P. acanthophyllus* against human cancer strains at 50 μM concentration (HCT, human colon cancer; MCF, human breast cancer).

According to studies on flavonoid structure–activity relationship (SAR), some substituents may improve biological activities. In general, elements such as the double bond between C2 and C3 carbons conjugated with the C4 carbonyl group confer molecular planarity necessary for delocalization of electrons. The catechol group in ring B is another contributor.^31,47,48^

In relation to antitumoral activity, the dihydroxyl group attached in C5 and C7 or even in C6 carbons is a positive contributor. In the case of the methoxy group, the polymethylation in ring A proved to be beneficial, but mainly when this substitution is in the C8 carbon. Also, the presence of the hydroxyl group in C3 carbon proved be a positive factor.^48^ The glycoside substitution in C3 carbon tends to decrease antitumor activity, due to the increase in molecular polarity, given the hydrophobicity of the cell membrane, in addition to the steric blocking caused by the size of the substituent.^31^ Figure 4A summarizes these positive and negative factors that are described.

**Figure 4:**
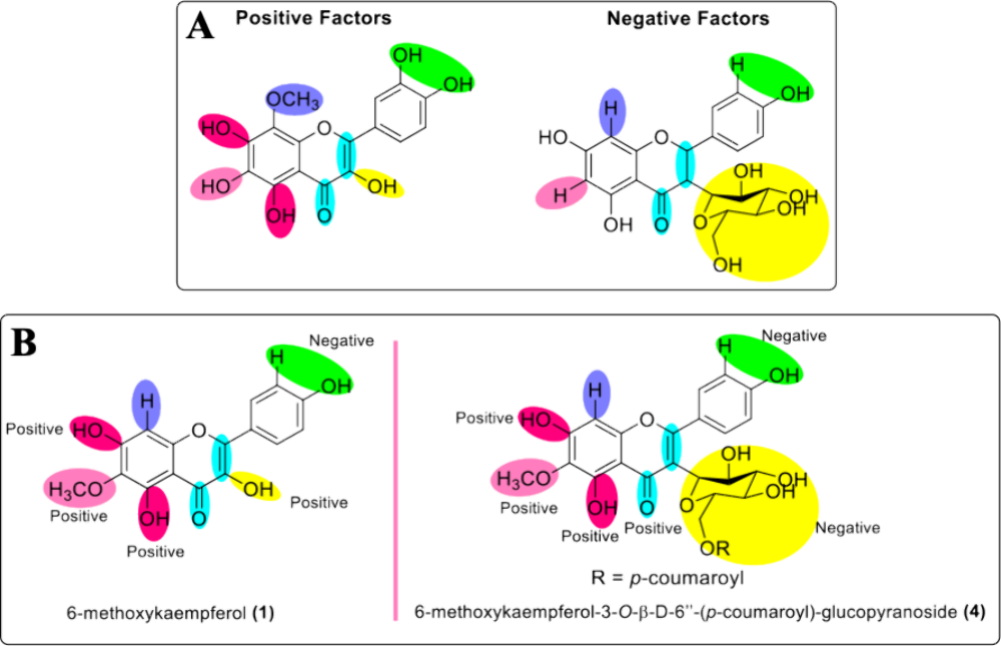
(A) Positive and negative substituents for antitumor activity; (B) positive and negative effects of compounds **1** and **4** substituents for antitumor activity.

The isolated compounds **1** and **4**, as all flavonols, have a double bond between C2 and C3 carbons conjugated to the carbonyl group in C4 and the dihydroxyl group in C5 and C7 carbons, which are positive factors for antitumoral activity. In fact, 6-methoxykaempferol (**1**) was slightly more active than compound **4**, but as mentioned before, both compounds only presented residual cytotoxicity (lower than 30% at 50 μM). Nonetheless, in general, factors to be considered for the low inhibition of tumor cells are the presence of the methoxyl group in C6 carbon and the absence of the hydroxyl group on C3′ carbon, fundamental to forming the catechol group in ring B. Compound **4**, in addition to the presence of methoxy at C6 carbon, also has the glycoside group linked to C3 carbon, probably explaining the lower observed cytotoxic activity (Figure 4B).

#### Evaluation of Antimicrobial Activity

The antimicrobial evaluation was performed for compounds **1**, **4,** and **5**, with concentrations ranging from 7.8 to 1000 μg mL^–1^. The colorimetric method of microdilution in broth with TTC was used against Gram-positive *Staphylococcus aureus* and Gram-negative *Escherichia coli* strains. The results obtained from the MIC are shown in Table 1.

**Table 1:** Minimum Inhibitory Concentration (MIC, μg mL^–1^) of Compounds **1**, **4**, and **5** against Strains of *S. aureus* and *E. coli*

	MIC/μg mL^–1^	
samples	*S. aureus* (NEWP0023)	*E. coli* (NEWP0022)
**1**	250	≥250
**4**	≥500	≥250
**5**	≥500	≥250
gentamicin	≤0.5	≤0.5

The antimicrobial activity for pure molecules can be considered significant when it has MIC values below 10 μg mL^–1^, moderate when MIC values are between 10 and 100 μg mL^–1^, and weak when above 100 μg mL^–1^.^49^ Thus, the evaluated compounds showed weak or inactive antimicrobial activity. Compound **1** showed enhanced activity against the *S. aureus* strain, and all evaluated compounds showed MIC values greater than 250 μg mL^–1^ for the *E. coli* strain.

According to studies done on the SAR of flavonoids in relation to antimicrobial activity (Figure 5A), the 4-oxo group in conjunction with the double bond of the C2=C3 carbons, the catechol group in the B ring, and the presence of hydroxyl attached to the C3 carbon contribute positively to activity. Furthermore, the presence of sugar and hydroxylation residues at carbons C5 and C7 is also a positive factor. However, the number of hydroxyl groups has a negative influence, as they result in lower hydrophobicity, with consequent obstruction of the flavonoid–biological membrane interaction.^31^

**Figure 5:**
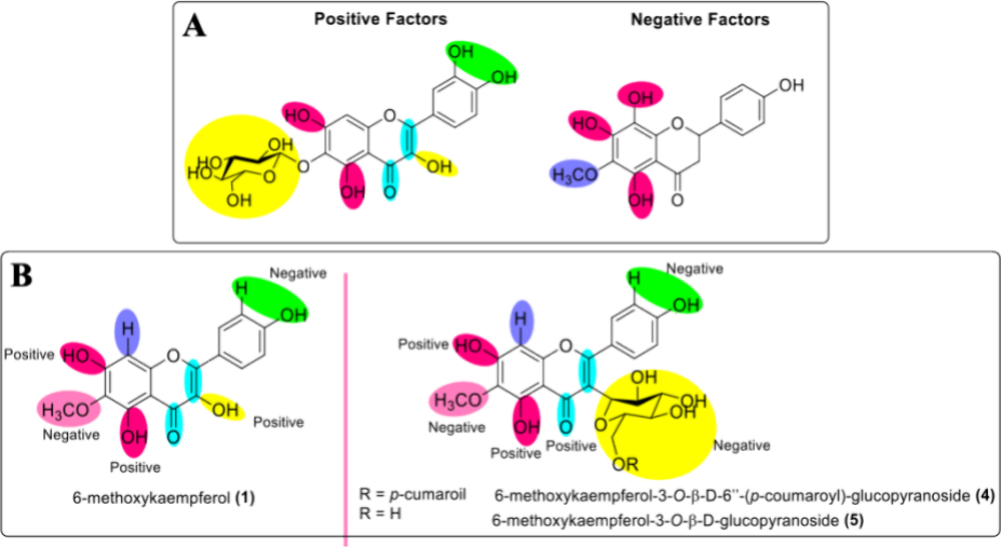
(A) Substituents with positive and negative effects on antimicrobial activity; (B) positive and negative effects of **1**, **4**, and **5** substituents on antimicrobial activity.

The two isolated glycosylated flavonoids showed decreased antimicrobial activity compared to the aglycone analogue (**1**) against the *S. aureus* strain. It is also found that the sugar residues are attached to the C3 carbon. This data suggests that the bonding of the hydroxyl to the C3 carbon tends to contribute more effectively to this activity than the presence of the glycoside in the same position. Furthermore, the fact that the three flavonoids have a methoxy group in their structure may have contributed to the low antimicrobial activity of these compounds. Figure 5B shows the positive and negative data for compounds **1**, **4**, and **5**.

#### Evaluation of the Antiviral Activity

##### Target-Based Assays

An initial screening of the isolated compounds was performed as the first step in evaluating the antiviral activity. In this step, the fluorescence produced by obtaining 7-amino-4-methylcoumarin (AMC) from the reaction between NS2B-NS3pro and substrate BZ-NKRR-AMC was measured. Compounds **1**, **3**, **4**, **5**, and **6** were tested at a concentration of 10 μM. The compounds showed inhibitory activities between 60 and 90% (Figure 6). Compound **4** showed inhibitory activity of the NS2B-NS3pro complex greater than 80% at 10 μM and was selected for IC_50_ value determination. The concentration capable of inhibiting 50% of the NS2B-NS3pro activity (IC_50_) was determined by OriginPro 9.0 software, which indicated that compound **4** presented an IC_50_ of 1.95 μM (Figure 6).

**Figure 6:**
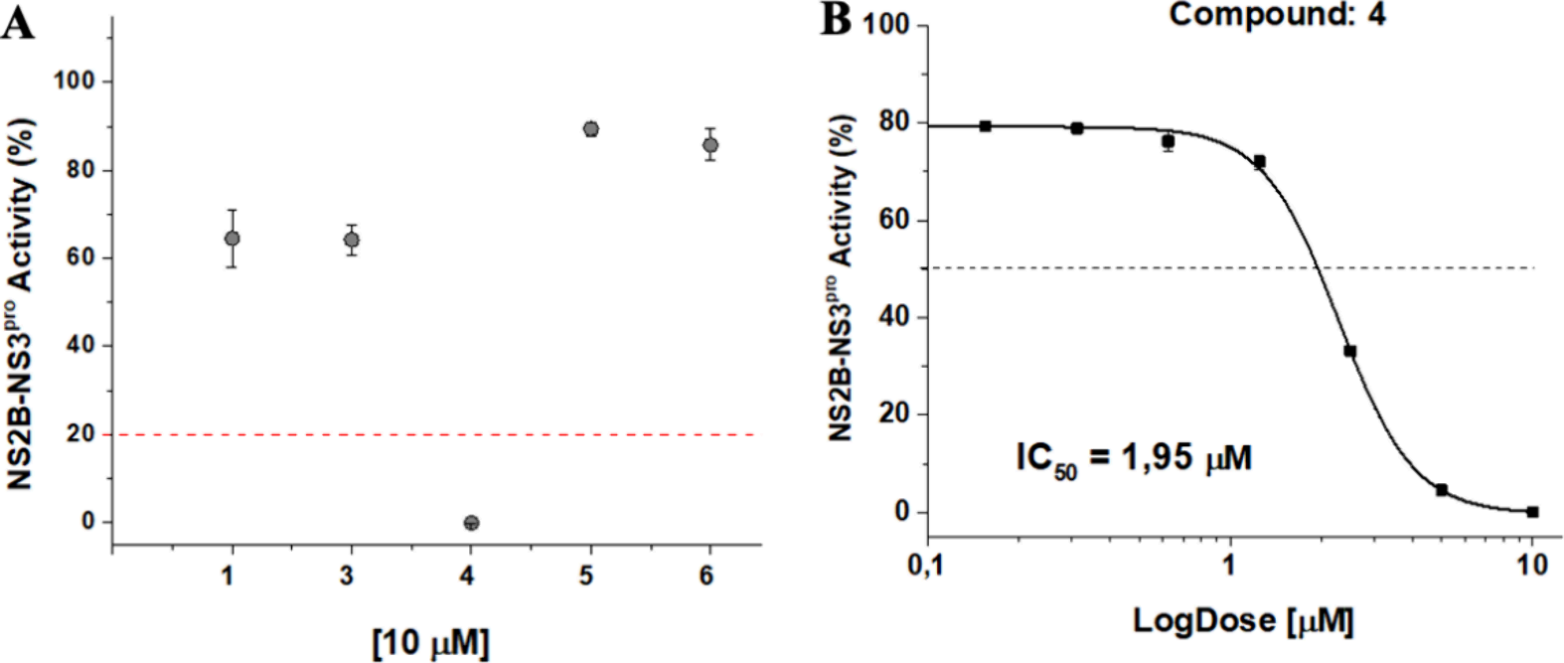
(A) Initial screening based on the relative activity of NS2B-NS3pro by compounds at 10 μM. The assay was performed in duplicate, and aprotinin was used as a positive control. The error bars represent the standard deviation. (B) Inhibitory concentration curve (IC_50_) of compound **4**. The assay was performed in duplicate, and aprotinin was used as a positive control. Error bars represent the standard deviation.

##### Molecular Modeling

To gain deeper insight into the inhibitory activity of compound **4**, we conducted molecular modeling to examine its binding within the NS2B-NS3pro binding site. The resulting model revealed that the inhibitor occupies the binding cavity, establishing favorable interactions with key residues in the binding site, including the catalytic triad Ser135, His51, and Asp75.^50^ Additionally, compound **4** forms hydrogen bonds with the side-chain carbonyl group of Asn152 and the main-chain atoms of Gly153 in the nonstructural protein NS3, as well as the main-chain atoms of Phe84 in cofactor NS2B (Figure 7). Notably, the model also shows that the phenolic group of the *p*-coumaroyl substituent is near the hydrophobic side chains of Tyr161 and V155 (NS3). These findings shed light on the molecular determinants contributing to the inhibitory potency of compound **4**.

**Figure 7:**
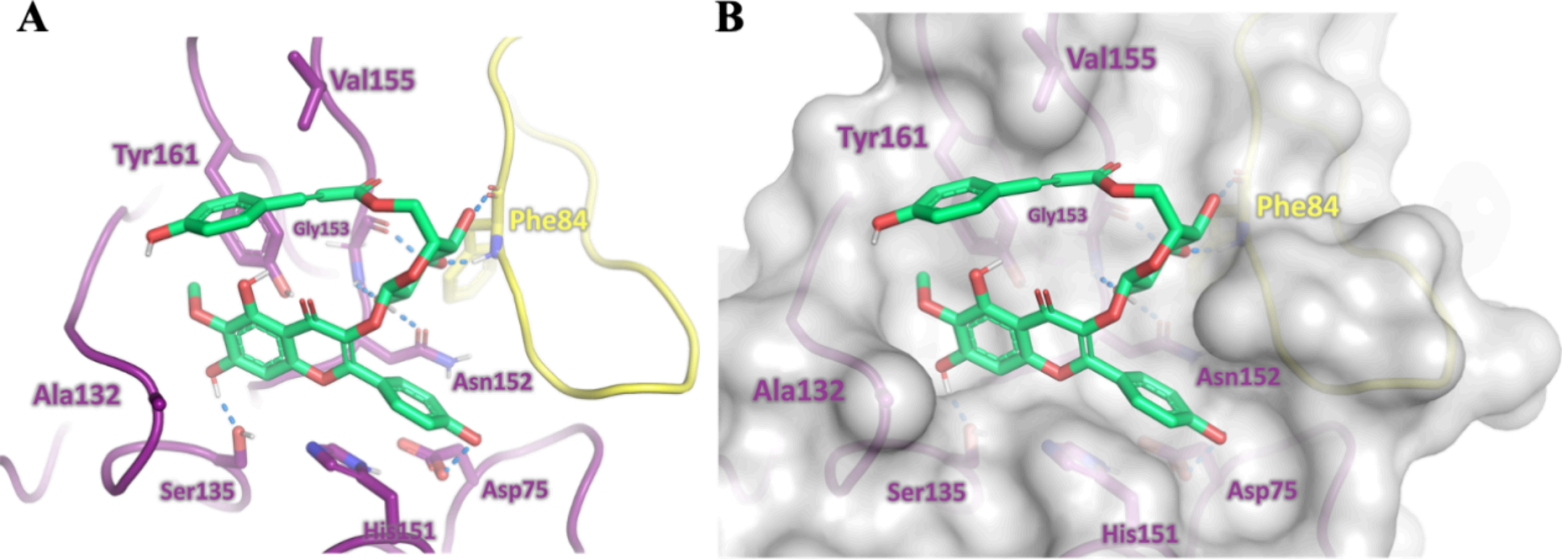
Modeled binding mode of compound **4** (green) to ZIKV NS2B-NS3 protease. (A) Structure of the cofactor NS2B is shown in yellow and that of NS3 in magenta. Polar contacts are presented as blue dashed lines. (B) Surface model of the ZIKV NS2B-NS3 protease.

##### Phenotypic Assays

RLuc activity and cell viability assays were performed for compound **4**. The compound did not inhibit the activity from Rluc, keeping its signal at 100%. The compound was not toxic to BHK-21-RepZIKV_IRES-Neo, maintaining cell viability at 100%, as shown in Figure 8.

**Figure 8:**
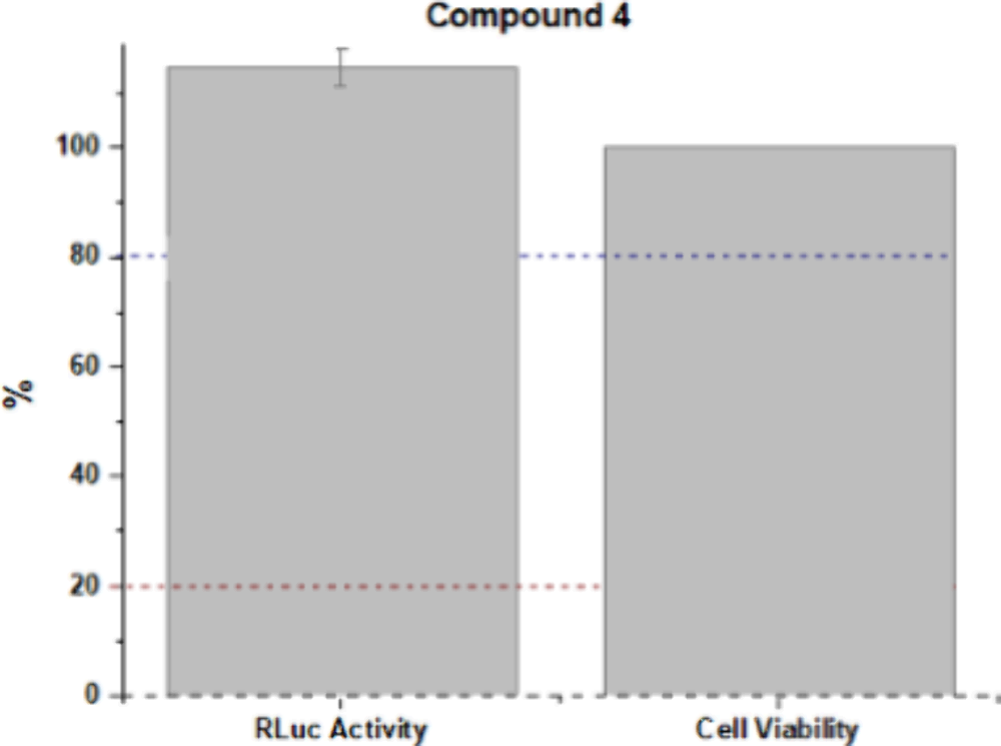
Evaluation of the relative inhibition of ZIKV replication and cell viability by compound **4** in mammalian cells (Line BHK-21-RepZIKV_IRES-Neo). The test was performed in duplicate, and NITD008 was used as a positive control. The error bars represent the standard deviation.

Compound **4**, which has a polar property, may not have been able to cross the cell membrane since it consists of a lipid bilayer, which is characterized by nonpolar properties. Furthermore, compound **4** may also have failed to interact with NS2B-NS3pro in the replication complex. It should be noted that the information presented above is hypotheses, and additional tests are required for confirmation.

The SAR of compound **4** indicates why it is the most active in relation to the other compounds tested against ZIKV. According to the literature,^51,52^ the most important factor in this activity is the catechol group in the B ring. It was found that no tested flavonoid has this group; however, they all have a hydroxyl. The double bond between C2 and C3 conjugated to the 4-oxo group is also important but not essential. However, the presence of the methoxy group linked to 4′-OCH_3_ decreases the activity, which justifies compound **4** having shown better activity in relation to compound **2**. The presence of sugar linked to the C3 carbon increases the activity; however, the binding of the sugar to the C7 carbon causes it to decrease, especially when bulky groups are added, justifying the greater activity of compound **4** compared to compound **6**. Compounds **4** and **5** have the same advantages and disadvantages as compound **4**; however, compound **4** presents the coumaroyl group linked to the sugar, which previous studies had shown to be advantageous for activity against ZIKV.^53^

## 4 Conclusions

The pantropical family Eriocaulaceae comprises about 1200 species, divided into 10 genera. *Paepalanthus*, the second largest genus of the family, with about 400 species, has only 26 studied species. Many compounds isolated from *Paepalanthus* species had their in vitro biological activities tested, and activities such as mutagenic, antimicrobial, antioxidant, anti-inflammatory, and antitumor were confirmed. Chemical investigation of the *P. acanthophyllus* and *P. bromelioides* capitula resulted in the isolation of seven compounds. Compound 6-methoxykaempferol-7-*O*-β-d-glucopyranoside (**6**) was isolated for the first time in the literature. Compounds 3′,4’-dimethoxyphysetin (**2**) and *p*-hydroxybenzoic acid (**3**) were reported for the first time in the family, and compounds 6-methoxykaempferol (**1**) and 6-methoxykaempferol-3-*O*-β-D-6″-(*p*-coumaroyl)-glucopyranoside (**4**) were reported for the first time in the species. Compounds 6-methoxykaempferol-3-*O*-β-d-glucopyranoside (**5**) and paepalantine (PbD-01) were previously isolated from *P. acanthophyllus* and *P. bromelioides* species. Compounds **1** and **4** presented weak cytotoxicity in both colon and breast tumor cells, with compound **1** being slightly more active when compared to compound **4**, which can be explained by structural differences. The evaluation of the antimicrobial activity showed better activity of compound **1** against the Gram-positive strain *S. aureus* and of compounds **4** and **5** against the Gram-negative strain *E. coli* . The evaluation of antiviral activity was carried out with compounds **1**, **3**, **4**, **5**, and **6**. Only compound **4** inhibited the NS2B-NS3pro enzyme in the low micromolar range in enzymatic assays. Docking studies shed light on the molecular determinants contributing to the inhibitory potency of compound **4**. Despite this, the compound did not show inhibition when evaluated in phenotypic assays with the ZIKV replicon. *P. acanthophyllus* species was interesting in the chemical scope, mainly its CH_2_Cl_2_ fraction, where its chemical profile obtained by HPLC-UV showed a quantitative variety of compounds. Based on this phytochemical study, the species shows promise for new phytochemical studies and isolated compounds for the evaluation of other biological activities.

## Experimental Section

### Materials and Reagents

Isolation of compounds by HPLC was performed using a preparative Zorbax SB-C18 column (250 × 21.2 mm i.d., 7.0 μm, Agilent Technologies). Silica gel 60 ACC (Agilent Technologies 70–90 mesh) or Sephadex LH-20 (GE Healthcare Bio-Sciences AB) was used as the stationary phase in classical chromatography (CC). Thin-layer chromatography (TLC) analyses were performed in silica gel plates (Macherey-Nagel, F254, 20 cm × 20 cm × 0.20 mm; F254, 20 cm × 20 cm × 0.25 mm).

The chemicals used were acetonitrile (CH_3_CN) and methanol (MeOH) HPLC grade from Panreac Quimica (Barcelona Spain), dichloromethane PA and ethyl acetate (EtOAc) PA from Isofar (Niterói, RJ, Brazil) purified by distillation, EtOAc, vanillin PA, and hexane PA from Neon (Suzano, SP, Brazil); EtOAc HPLC grade and glacial acetic acid PA from Vetec (Duque de Caxias, RJ, Brazil), deuterated dimethyl sulfoxide (DMSO) from Sigma-Aldrich (St. Louis, Missouri, USA) with a peak at δ 2.50 ppm, deuterated methanol (CD_3_OD) from Cambridge Isotope Laboratories (Tewksbury, MA, USA) with a peak at δ 3.30 ppm, methanol PA from Synth (Diadema, SP, Brazil), trifluoroacetic acid from Anidrol (Diadema, SP, Brazil), ferric chloride PA, potassium hydroxide PA, and sulfuric acid PA from Dinâmica (Diadema, SP, Brazil), and ethanol (EtOH) PA from Tedia (Rio de Janeiro, RJ, Brazil).

For the biological activities, we used aprotinin, fetal bovine serum (FBS) (Thermo Fisher Scientific, Waltham, MA, USA), cell counting kit-8 (Sigma-Aldrich, St. Louis, Missouri, USA), doxorubicin (Sigma-Aldrich, St. Louis, MO, USA), Dulbecco’s modified Eagle's medium (DMEM) (Thermo Fisher Scientific, Gibco, Kennett Square, PA, USA), gentamicin (Sigma-Aldrich, St. Louis, Missouri, USA), kit Renilla luciferase Assay System (Promega, Madison, WI, USA), methylthizol tetrazolium (Thermo Fisher Scientific, Waltham, MA, USA), Mueller–Hinton agar (Sigma-Aldrich, St. Louis, Missouri, USA), Mueller–Hinton broth (Kasvi, São José dos Pinhais, PR, Brazil), *Staphylococcus aureus* (NEWP0023, Newprov, Pinhais, PR, Brazil), *Escherichia coli* (NEWP0022, Newprov, Pinhais, PR, Brazil), triphenyl tetrazolium chloride (TTC, Sigma-Aldrich, St. Louis, Missouri, USA), Renilla luciferase (Promega, Madison, WI, USA), Roswell Park Memorial Institute medium (RPMI) (Thermo Fisher Scientific, Waltham, MA, USA), and streptomycin penicillin (Thermo Fisher Scientific, Waltham, MA, USA).

### Equipment

The following equipment was used: air heater (Ethik Technology, Vargem Grande Paulista, SP, Brazil), analytical balance model AUY220 (Shimadzu, Kyoto, Japan), Balance model 9094 c/4 (Toledo, São Bernardo do Campo, SP, Brazil), blender (Arno, Embu das Artes, SP, Brazil), heating mantles model 302 (Fisatom, Perdizes, SP, Brazil), Fourier transform ion cyclotron resonance mass spectrometry (FT-ICR) MS model 9.4T Solarix (Bruker Daltonics, Billerica, MA, USA), high-performance liquid chromatography model 1260 Infinity (Agilent Technologies, Santa Clara, CA, USA) composed of a prebinary pump (G1361A), two valves (G1170, 1290 Infinity), manual injection, automatic collector (G1364B), MWD (G1365D) detector, incubator (Panasonic, Osaka, Japan), MCF-500 McFarland turbidimeter (MS Tecnopon, Piracicaba, SP, Brazil), medium-pressure liquid chromatography (MPLC) model C-810 Flash (BUCHI, New Castle, NE, USA) equipped with a control unit, manual injector, four solvent channels, gradient valve, one pump, fraction collector, and ultraviolet detector, microplate ultraviolet spectrophotometer (Thermo Fisher Scientific, Waltham, MA, USA), nuclear magnetic resonance model INOVA 400 MHz (Varian, now Agilent technologies, Santa Clara, CA, USA), rotary evaporator model R-100 (BUCHI, New Castle, NE, USA), composed of a heating bath (B-100), vacuum pump (V-100) with a pressure controller (I-100), and ultrathermostatic bath (Solab-SL 152), SpectraMax 384 Plate Reader and SpectraMax i3Multimode Detection Platform (Molecular Devices, San Jose, CA, USA), turbidimeter model MCF-500 McFarland (MS Tecnopon, Piracicaba, SP, Beazil), ultrasound bath model Ulltra Cleaner 1600 (Ultronic-Unique, Indaiatuba, SP, Brazil), and ultraviolet light chamber (λ = 254 and 366 nm) (Camag, Muttenz, Switzerland).

### Software

Software ChemDraw (PerkinElmer Informatics) was used to draw the chemical structures, Bruker Compass DataAnalysis (Bruke Daltonik GmbH) to open the mass spectra, OriginPro 9.0 (OriginLab) to do mathematical calculations and graphical plotting of the data of antiviral activity, Prism 5 (GraphPad Software) to do mathematical calculations and graphical plotting of the data of cytotoxic activities, MestReNova (MestreLab Research S. L) to analyze the NMR spectra, and the software package SYBYL X (Tripos) for molecular modeling.

### Plant Material

*P. acanthophyllus* capitula were collected at “Chapada dos Veadeiros” (13°41′6″S, 47°28′14″W), state of Goiás, Brazil, on July 21, 2016. The specimens were identified by Dr. Marcelo Trovó Lopes de Oliveira of the Federal University of Rio de Janeiro (UFRJ). A voucher specimen (VIES 025706) was deposited in the Federal University of Esprito Santo UFES herbarium. *P. bromelioides* was collected at “Serra do Cipó” MG on October 17, 2019. The identification of the species was carried out by researcher Celso Lago-Paiva, and its specimen is deposited in the UFES Herbarium (VIES 45870).

### Extraction and Isolation

#### Paepalanthus Acanthophyllus

*P. acanthophyllus* capitula were dried (2.0 kg) in an air heater at 40 °C, and after the milling, they were submitted to maceration with MeOH. The crude extract was resuspended in MeOH/H_2_O (3:1, v/v) and partitioned with hexane (7.0 g), CH_2_Cl_2_ (20.0 g), and EtOAc (25.0 g), with 2.5 L of each eluent. All fractions were dried in a rotary evaporator.

The CH_2_Cl_2_ fraction (∼18.0 g) was submitted to MPLC using silica gel (70–90 μm) as the stationary phase and the solvents hexane, EtOAc, and MeOH acidified with 0.1% acetic acid as the mobile phase at a flow rate of 20 mL·min^–1^. In this experiment, 16 subfractions were obtained, which were combined according to the similar chemical profile by TLC. Subfractions 10–44 (5.2645 g) showed a major compound after evaluation by TLC and staining with sulfuric vanillin. The subfraction was subjected to CC, packed with silica gel (38.0 cm × 5.0 cm × 2.5 cm), and eluents hexane, EtOAc, and MeOH were used in increasing order of polarity. The separation afforded 16 subfractions. Fractions 46–69 and 100–113 were studied. Subfractions 46–69 (671.8 mg) showed a reddish color and gave a yellow precipitate. This was separated from the supernatant and washed with EtOAc (HPLC grade). The pure precipitate (55.4 mg) was coded as compound **1**. The reddish subfractions 100–113 (498.1 mg) also gave rise to a yellow precipitate. The procedure for separating the supernatant and cleaning the precipitate was the same as that described above. The pure precipitate (32.9 mg) was coded as compound **2**.

Compound separation of the EtOAc fraction was performed by CC (35.0 × 5.2 × 2.5 cm) with hexane, EtOAc, and MeOH in increasing order of polarity. The EtOAc/hexane (7:3, v/v) system yielded a pure (154.4 mg) and an impure (901.5 mg) compound **1** obtained in a gathered fraction 3–6. The impure fraction 5 (16.4 mg) was submitted to preparative HPLC-UV, with an injected volume of 400 μL, at a flow rate of 12.0 mL min^–1^. The mobile phase consisted of 90% ultrapure water (eluent A) and 10% acetonitrile (eluent B), in a gradient mode, until 100% acetonitrile in 35 min. This procedure afforded compounds **1** (3.4 mg) and **3** (1.8 mg).

Subfractions 17–56 (3.1191 g) were subjected to Sephadex LH-20 gel filtration (88.7 × 3.0 × 1.8 cm) using MeOH as an eluent. The procedure resulted in 225 aliquots being reunited into 40 subfractions, after analyzing their similarity in TLC. The resulting subfractions 44–48, 49–54, and 64–75 were fractionated into columns. Subfractions 44–48 (85.1.0 mg), solubilized in 900 μL of MeOH, were subjected to purification by HPLC on a preparative column. The mobile phase was composed of ultrapure water (A) and 10–40% CH_3_CN (B) until 30 min, 50% CH_3_CN until 35 min, and 50–100% CH_3_CN until 45 min, with a flow rate of 7 mL min^–1^. Injections were performed in a volume of 100 μL, and nine subfractions were obtained. Subfraction 1 (2.2 mg) was analyzed by ^1^H NMR and coded as compound **3**. Subfractions 49–54 (70.0 mg), solubilized in 1.1 mL of MeOH, were subjected to purification by HPLC on a preparative column. The mobile phase was composed of ultrapure water (A) and 10–50% CH_3_CN (B) until 15 min and 50–100% CH_3_CN until 25 min, with a flow rate of 7 mL min^–1^. Injections were performed in a volume of 100 μL. In this procedure, 15 subfractions were obtained, but only subfraction 4 (4.1 mg), coded as compound **4**, was shown to be pure after analysis by ^1^H NMR. Subfractions 64–75 (170.6 mg) were subjected to adsorption liquid chromatography and packed with silica gel (75 × 2.5 × 0.2 cm). Hexane and EtOAc solvents formed the initial mobile phase, used in a 1:1 ratio. The eluent system inserted was in increasing order of polarity up to 100% ultrapure water. This procedure yielded 17 subfractions, reunited according to the chemical profile on TLC. Subfractions 26–55 (13.3 mg) were subjected to NMR analysis and were shown to be compound **1**.

Subfractions 101–103 (333.9 mg) were also purified by preparative HPLC-UV with an injected volume of 50 μL at a flow rate of 7.0 mL min^–1^. The mobile phase consisted of ultrapure water (eluent A) and 32–64% CH_3_CN (eluent B) until 40 min and 64–100% CH_3_CN until 60 min. Each mobile phase was acidified with TFA 0.01%.^7^ This procedure yielded compound **4** (34.8 mg).

Subfractions 104–111 (959.0 mg) were submitted to adsorption liquid chromatography and packed with silica gel (97.3 × 2.5 × 0.9 cm), and hexane and EtOAc solvents formed the initial mobile phase, used in a ratio of 1:1. The eluent system was added in increasing order of polarity, up to MeOH:ultrapure water (1:1, v/v). The procedure gave rise to 15 subfractions. The 139–189 subfractions (301.5 mg) obtained in this procedure were fractionated on a column packed with silica gel (80.0 × 2.5 × 0.25 cm). Fractionation of subfractions 139–189 started with EtOAc and hexane solvents, in a 9:1 ratio. The eluent system was added in increasing order of polarity, up to 100% MeOH. The procedure gave rise to eight subfractions. Subfractions 106–120 (59.7 mg) afforded compound **4**.

The oily subfractions 122–127 (∼7.1234 g) were subjected to adsorption liquid chromatography and packed with silica gel (40.0 × 5.0 × 0.7 cm). The eluent system started with EtOAc 100% to MeOH:ultrapure water (1:1, v/v), in increasing order of polarity. The procedure resulted in 11 subfractions. After dividing subfractions 28–36 (1.2357 g), 100.0 mg of it was sent for exclusion chromatography using Sephadex LH-20 (19.4 cm × 2.0 cm) and MeOH as the mobile phase, resulting in 18 aliquots, grouped into nine subfractions. Among them, subfractions 5 and 8–9 were studied. Subfraction 5 (16.7 mg) yielded compound **5**, which appeared as a single orange spot when revealed with sulfuric vanillin. This compound was analyzed by NMR and mass spectrometry. Subfractions 8–9 (6.4 mg) were separated by Sephadex LH-20 (32.0 cm × 2.0 cm) exclusion chromatography using MeOH as the mobile phase, resulting in five aliquots. Subfraction 5 (2.9 mg), encoded as compound **6**, appeared as a single orange spot when stained with sulfuric vanillin and was analyzed by NMR spectroscopy.

#### Paepalanthus Bromelioides

The flower heads (1.2 kg) of the species were collected dry and subjected to maceration using MeOH as the extracting solvent. The plant residue was subsequently extracted with CH_2_Cl_2_, obtaining 8.0 g of crude extract that was subjected to fractionation by MPLC. The CH_2_Cl_2_ extract from *P. bromelioides* (50.0 mg) was submitted to preparative TLC with an elution system composed of hexane:EtOAc (7:3, v/v). This procedure yielded the compound coded as PbD-01 (5.7 mg).

### Biological Assays

Biological assays were carried out according to the mass availability of the compounds. Therefore, compounds **3** and **6** did not have all of the biological activities proposed in this article.

#### Cytotoxic Activity Evaluation

In vitro cytotoxic activities of isolated compounds **1** and **4** were evaluated in HCT-116 (human colon cancer) and MCF-7 (human breast cancer) according to the literature.^39^ Both cells were chosen due to their importance. HCT-116 cells are characterized by their high oncogenic aggressiveness,^54^ and MCF-7 is one of the most studied human cancer cell lines in the world and has an important impact on breast cancer research.^55^ Furthermore, according to Santos, breast and colon cancers are the most common cancers in Brazil.^56^ HCT-116 cells were grown in RPMI, and MCF-7 (breast carcinoma) cells were grown in DMEM. The media were supplemented with 10% FBS and 1% penicillin and streptomycin. Cells were kept in an incubator with 5% CO_2_ at 37 °C.

For the MTT assay, the cells were seeded on 96-well plates at a density of 1 × 10^4^ per well with 200 μL of culture medium. After 24 h, the samples were added at a concentration of 50 μM. After the treatment, the plates were incubated for 72 h. DMSO was used as a negative control (0.05%), and the antineoplastic doxorubicin (0.00064–10 μM) was used as the positive control.

The culture media were replaced by fresh media containing MTT solution at 0.5 mg mL^–1^, 3 h before the end of the experiment. The MTT solution was removed at the end of 72 h, and the formazan product was solubilized in 150 μL of DMSO. After 72 h, the MTT solution was removed, and 150 μL of DMSO was used to solubilize the formazan product.

The absorbance measurements were performed at 540 nm. The IC_50_ values and their 95% confidence interval were calculated by sigmoidal nonlinear regression using GraphPad Prism 5 software.

#### Antimicrobial Activity Evaluation

The antimicrobial activity evaluation method was performed according to the literature.^57^ Compounds **1**, **4**, and **5** were analyzed against two standard bacterial strains, *Staphylococcus aureus* (NEWP0023) and *Escherichia coli* (NEWP0022). These strains were chosen to represent some of the most common microbial pathogens that cause a significant threat to human health.^58–60^ *S. aureus* is a Gram-positive bacterial human pathogen, and *E. coli* was included as an example of a Gram-negative pathogenic bacterium. Plates with 96 wells were prepared by dispensing 100 μL of Mueller–Hinton broth into each well. Stock solutions were prepared of each compound in DMSO, and serial dilutions were made to reach a concentration ranging from 7.8 to 1000 μg mL^–1^ with the final volume completed to 100 μL. Gentamicin was used as a positive control at concentrations ranging from 60 to 0.5 μg mL^–1^, and DMSO was used as the blank.

The bacterial inoculum was an overnight culture grown in Mueller–Hinton agar and suspended in a sterile saline solution (0.45%) at a concentration close to 10^8^ CFU mL^–1^. This solution was diluted 1:10 in a saline solution, and an aliquot of 5 μL was added to each well.

The microdilution trays were incubated at 36 °C for 24 h. An aqueous solution (0.5%) of TTC was added (20 μL) to each well, and the trays were incubated at 36 °C for 2 h. Then, the color change was verified. It is observed that there is a change of color from colorless to red in the well that had bacterial growth.

Experiments were performed in triplicate. The result was expressed in MIC. MIC has the unit μg mL^–1^, and it is defined as the lowest concentration of each compound in which no color change occurred.

#### Antiviral Assays

In target-based assays, compounds **1**, **3**, **4**, **5**, and **6** were evaluated. The first step was the elaboration of the NS2B-NS3pro complex, which is a nonstructural protein (NS) responsible for viral replication; that is, it is the target for the development of antiviral drugs. The recombinant ZIKV protease corresponds to residues 45–96 of the NS2B cofactor linked to residues 1–177 of the NS3 protease domain by a glycine-rich spacer (linker) [G4SG4], and the hydrophobic transmembrane residues of NS2B were removed. The protease was expressed in Rosetta 2 (DE3) cells, purified by affinity and molecular exclusion chromatography, and stored at −80 °C, as described in the literature.^61^

The assay consisted of measuring the fluorescence obtained by the reaction between NS2B-NS3pro and the substrate BZ-NKRR-AMC, which resulted in the release of AMC.^61,62^ The reaction was performed in white 384-well plates using a reaction buffer containing 20 mM Tris pH 8.5, 10% glycerol, and 0.01% Triton X-100. Aprotinin (10 μM) was used as a positive control and DMSO 1% as a negative control. The assays were performed in duplicate.

The NS2B-NS3pro complex was added at a final concentration of 5 nM to the wells containing the reaction buffer. Then, the compounds were added to a final concentration of 10 μM. After 15 min of incubation at 37 °C, the BZ-NKRR-AMC substrate was added to a final concentration of 30 μM. The reaction was kept at a constant temperature of 37 °C, and the fluorescence was recorded every 1 min and 30 s for 20 min. The wavelengths used for excitation and emission were, respectively, 380 and 460 nm.

The percentage of inhibition was calculated from the slope of the line resulting from each measurement taken in the 20 min interval. The mean slopes of the aprotinin lines were used as 0%, and the mean slopes of the 1% DMSO lines were used as 100%.

The calculation of the inhibitory concentration (IC_50_) was performed for compounds that inhibited ≥80% of the enzyme activity. The compounds were added to the plate wells in duplicate, in serial dilution (factor 2), and the calculation of the relative activity was done as described below in the phenotypic assays. The concentration of compounds capable of inhibiting 50% of the activity of NS2B-NS3pro (IC_50_) was determined using OriginPro 9.0 software (Origin Lab).

In phenotypic assays, BHK-21-RepZIKV_IRES-Neo cells were maintained in DMEM containing 10% FBS and the aminoglycoside antibiotic G418 at a concentration of 500 μg mL^–1^. The compounds were further diluted in a culture medium to a final concentration of 10 μM. As a positive control for the assays, NITD008 was used, a potent flavivirus replication inhibitor.^63^

An assay based on *Renilla* luciferase activity^64,65^ was performed with approximately 2 × 10^4^ cells per well that were seeded in DMEM at 10% FBS in 96-well plates. After 16 h of incubation, the supernatant was discarded, and the DMEM was added again, but at 2% FBS, and the compounds were also added at a final concentration of 10 μM. After 48 h of incubation, the cells were lysed in 15 μL of *Renilla* luciferase Lysis Reagent lysis buffer for 15 min at room temperature (35 ± 2 °C) and under agitation. Then, 12 μL of the lysate was transferred to an opaque white 96-well plate containing 50 μL of *Renilla* luciferase substrate buffer (Rluc) and the luminescence; reading of the enzyme activity was performed in the SpectraMax i3Multimode Detection Platform equipment. 10 μM NITD008 compound was used as a positive control (100% inhibition) and 1% DMSO as a negative control (0% inhibition). The assay was performed in duplicate.

The proliferation assay was performed based on WST-8, using the Cell Counting kit-8.^65^ After incubation with the compounds, 10 μL per well of CCK8 solution was added, and the plates were incubated in a CO_2_ incubator at 37 °C for 2 h. Then, the absorbance was measured at 450 nm in the SpectraMax 384 Plate Reader. Cells in 1% DMSO were used as a negative control for the assay.

### Molecular Modeling

#### Docking Studies

The 3D structure of compound **4** was constructed using standard geometric parameters of the molecular modeling software package SYBYL X. The optimized conformation of the inhibitor was generated through energetic minimization utilizing the Tripos force field^66^ and the Powell conjugate gradient algorithm^67^ with the convergence criterion set at 0.05 kcal mol Å^–1^. Gasteiger–Hückel charges were applied to the structure.^68^ For molecular docking, compound **4** was docked into the NS2B-NS3 protease complex (PDB ID, 7OBV) (PDB ID, 7OBV)^69^ using Surflex-Dock.^70^ During the docking process, protein hydrogen and heavy atom movements were allowed, following default parameters. Specific residues such as histidine, glutamine, and asparagine within the binding site were manually checked for possible flipped orientation, protonation, and tautomeric states. The binding cavity of the NS2B-NS3 protease complex was defined using the protomol generation based on the 3D coordinates of the inhibitor MI-2248, experimentally determined in complex with the NS2B-NS3 protease^69^ (protomol parameters: proto_thresh 0.51 and -proto_bloat 4). The docking protocols were repeated 20 times, and the representative conformation for the inhibitor was selected based on the Surflex-Dock scoring function and visual inspection.

## Supplementary Material



## Notes

All NMR and HRMS spectral data of compounds **1–6** and PbD-01 (Figures S1–S32) (PDF)

## #

In memoriam: Lucas Almeida Oliveira—September 10, 2019.
