# Supplementary material for: Compounds Isolated from Paepalanthus spp. (Eriocaulaceae) and Their Evaluation in Antimicrobial, Cytotoxic, and Antiviral Assays
Source: ACS Omega. 2025 Jun 18;10(25):26403–14. doi: 10.1021/acsomega.4c11026 (PMC12223841; doi:10.1021/acsomega.4c11026)

*Supplementary information*

# Compounds isolated from *Paepalanthus* spp. (Eriocaulaceae) and their evaluation in antimicrobial, cytotoxic, and antiviral assays

Laysa Lanes Pereira Ferreira Moreira,[a] Lucas Almeida Oliveira†,[a] Raphael Conti,[a] Larissa Costa de Almeida,[b] Leticia V. Costa-Lotufo,[b] Ana Camila Micheletti,[c] Isabela Dolci,[d] Rafaela Sachetto Fernandes,[d] Glaucius Oliva,[d] Rafael Victorio Carvalho Guido,[d] Valdemar Lacerda Jr.,[a] Keyller Bastos Borges*,[e] and Warley de Souza Borges*[a]

[a] Departamento de Química, Universidade Federal Espírito Santo, Vitória, Espírito Santo, Brazil: laysalannes@hotmail.com; raphael.conti@gmail.com; valdemar.lacerda@ufes.br; warley.borges@ufes.br

[b] Departamento de Farmacologia, Universidade de São Paulo, São Paulo, São Paulo, Brazil: almeidalc@usp.br; costalotufo@usp.br

[c] Instituto de Química, Universidade Federal do Mato Grosso do Sul, Campo Grande, Mato Grosso do Sul, Brazil: anamicheletti@gmail.com

[d] Instituto de Física de São Carlos, Universidade de São Paulo, São Carlos, São Paulo, Brazil: isabela@estudante.ufscar.br; rafaela.fernandes@usp.br; oliva@ifsc.usp.br; rvcguido@usp.br

[e] Departamento de Ciências Naturais, Universidade Federal de São João del-Rei, Campus Dom Bosco, Praça Dom Helvécio 74, Fábricas, 36301-160, São João del-Rei, Minas Gerais, Brazil

keyller@ufsj.edu.br

† *In memoriam: Lucas Almeida Oliveira - 10 September 2019*

Correspondence:

Prof. Warley de Souza Borges, Ph.D., Programa de Pós-Graduação em Química, Centro de Ciências Exatas, Universidade Federal do Espírito Santo, Avenida Fernando Ferrari 514, Goiabeiras, 29075-910, Vitória, Espírito Santo, Brazil.

e-mail: warley.borges@ufes.br, Phone number: +55 27 4009 – 2908

Prof. Keyller Bastos Borges, Ph.D., Departamento de Ciências Naturais, Universidade Federal de São João del-Rei, Campus Dom Bosco, Praça Dom Helvécio 74, Fábricas, 36301-160, São João del-Rei, Minas Gerais, Brazil.

e-mail: keyller@ufsj.edu.br; Phone number: +55 32 3379 – 5163

6-methoxykaempferol (**1**): $^{1}H$ NMR (400 MHz, DMSO-$d_6$) δ 3.77 (*s*, 3H, 6-$OCH_3$), 6.55 (*s*, 1H, H-8), 6.93 (*d*, 2H, 8.0 Hz, H-3′/H-5′), 8.05 (*d*, 2H, 8.0 Hz, H-2′/H-6′); $^{13}C$ NMR (100 MHz, DMSO-$d_6$) δ 60.2 ($CH_3$, 6-$OCH_3$), 94.0 (CH, C-8), 103.6 (C, C-10), 115.7 (CH, C-3′/C-5′), 121.9 (C, C-1′), 129.8 (CH, C-2′/C-6′), 131.1 (C, C-6), 135.6 (C, C-3), 147.2 (C, C-2), 151.6 (C, C-9), 151.9 (C, C-5), 157.4 (C, C-7), 159.4 (C, C-4′), 176,3 (C, C-4).

**Figure S1**. $^{1}H$ NMR spectrum (400 MHz, DMSO-$d_6$) of compound **1**.

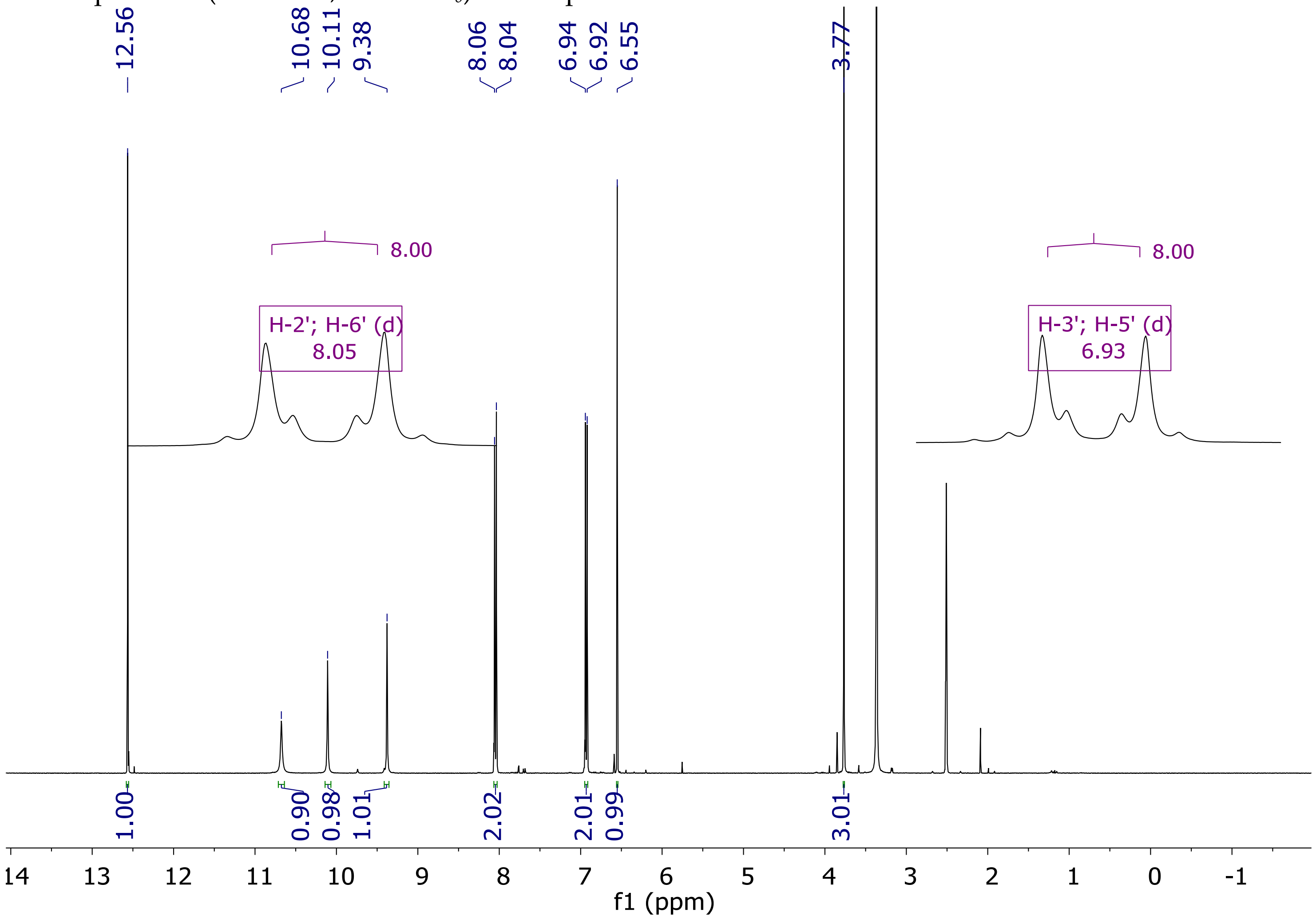

**Figure S2**. $^{13}$C NMR spectrum (100 MHz, DMSO-$d_6$) of compound **1**.

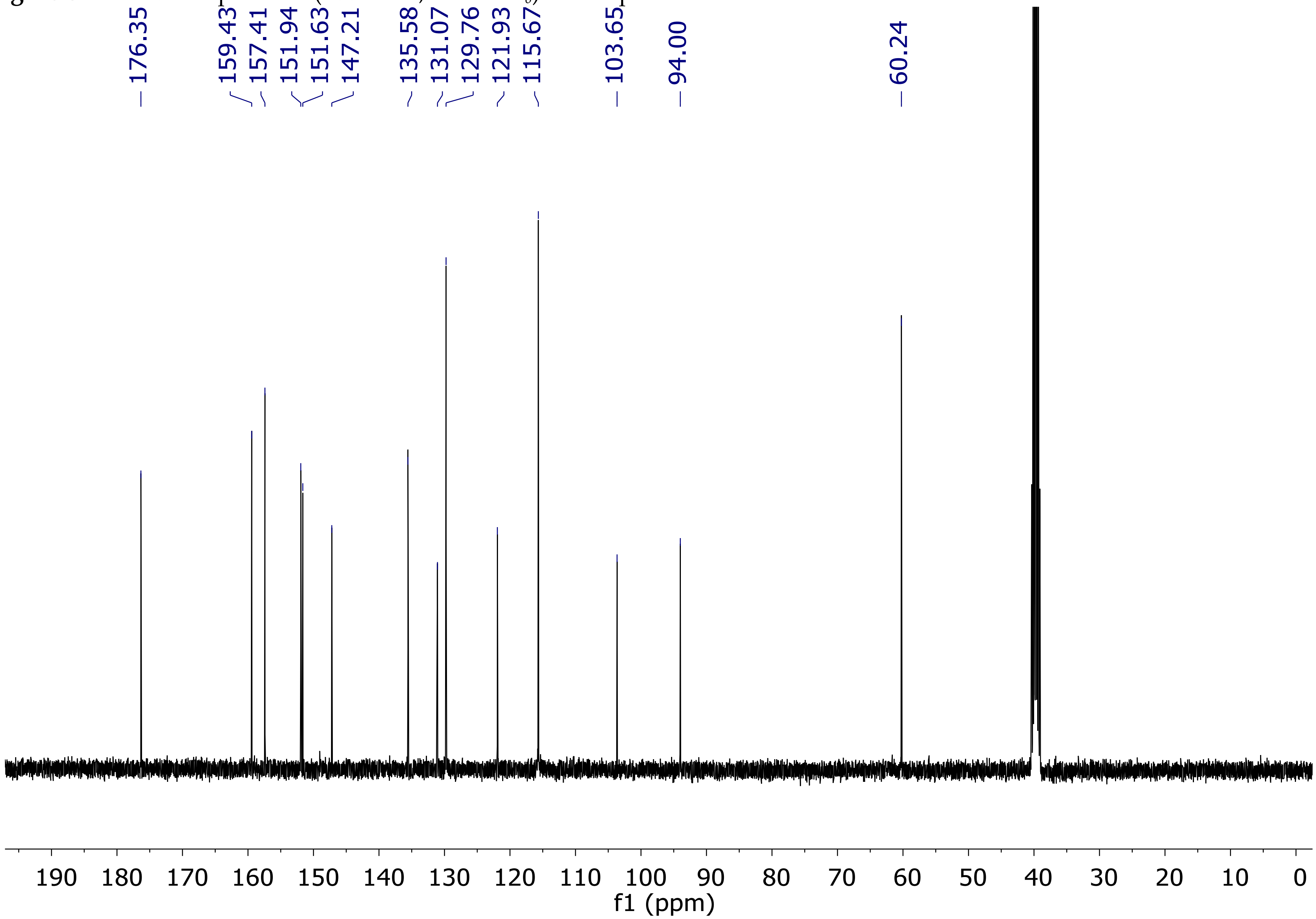

**Figure S3**. $^{1}H$ x $^{1}H$ (COSY) NMR spectrum (400 MHz, DMSO-$d_6$) of compound **1**.

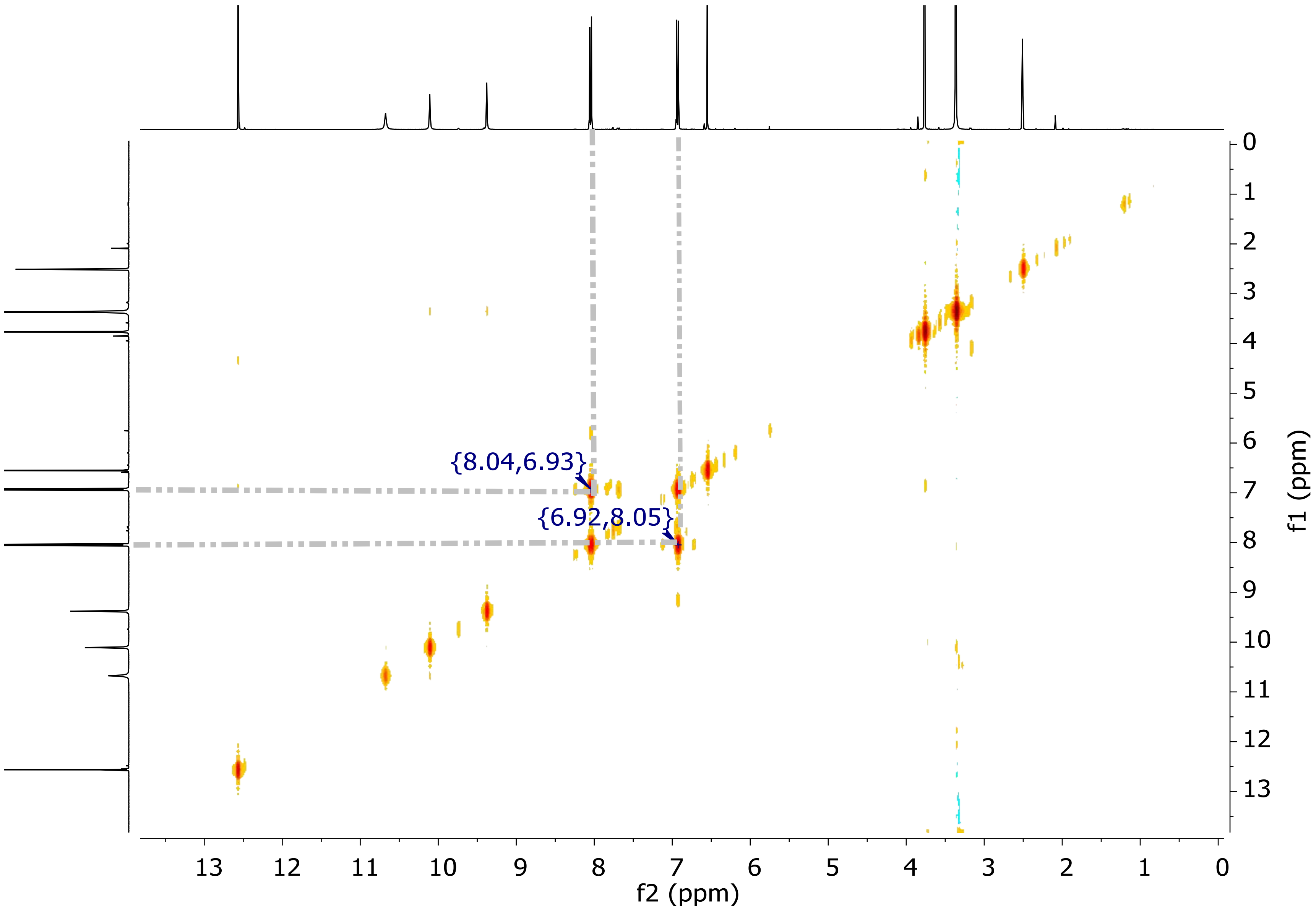

**Figure S4**. $^{1}H$ x $^{13}C$ (HSQC) NMR spectrum of compound **1**.

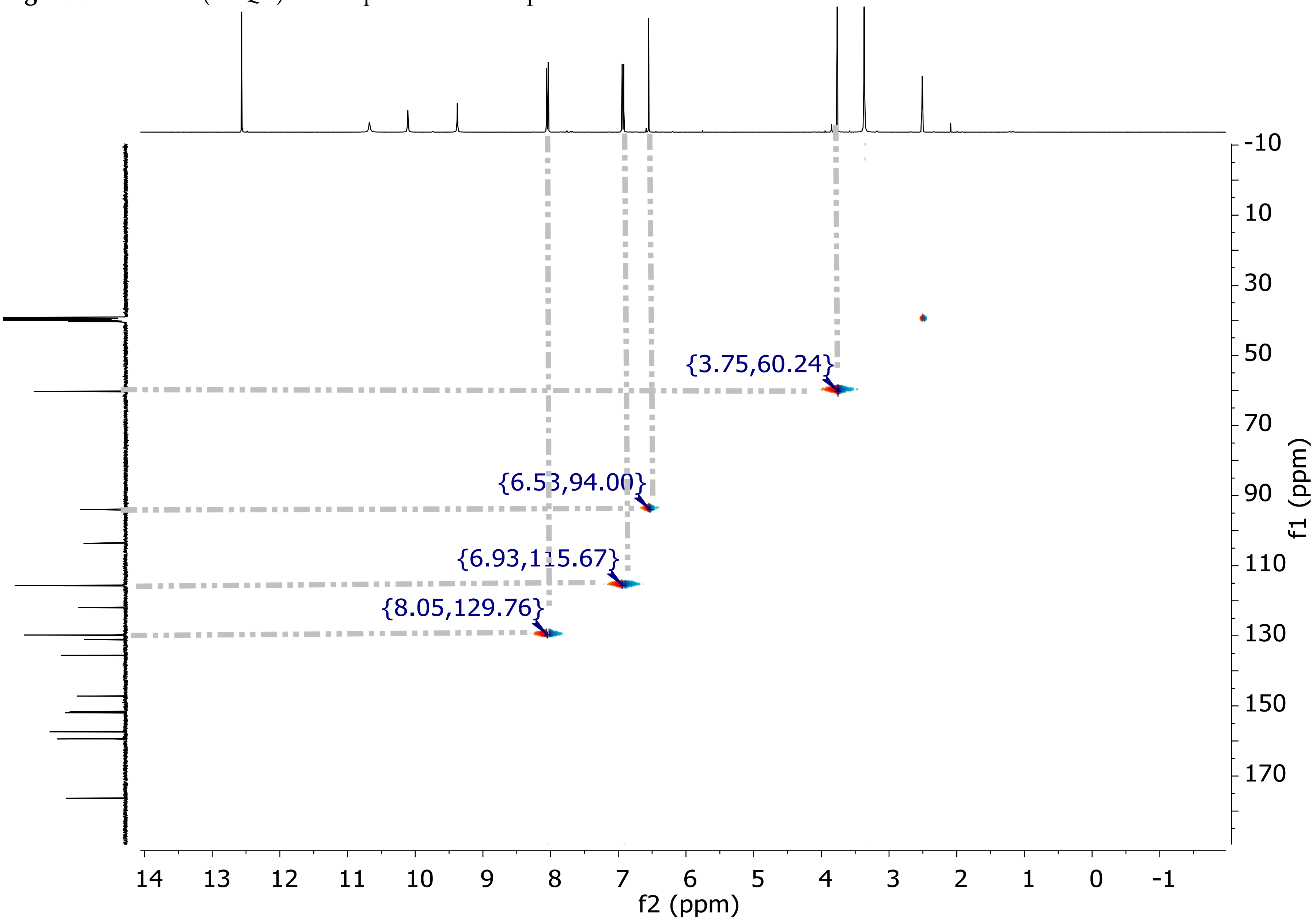

**Figure S5**. $^{1}$H x $^{13}$C (HMBC) NMR spectrum of compound **1**.

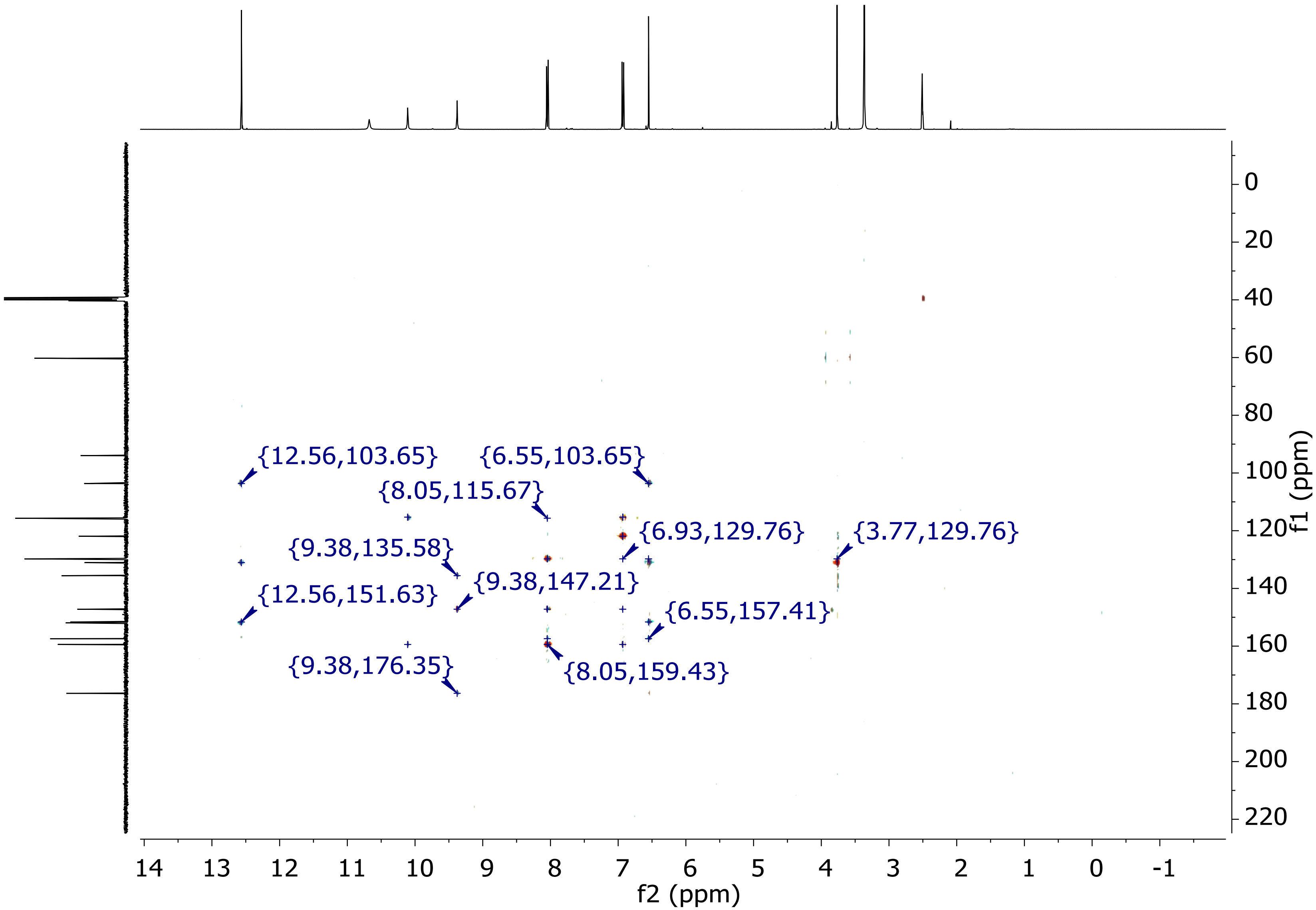

3′,4′-dimethoxyfisetin (**2**): $^1$H NMR (400 MHz, DMSO-$d_6$) δ 3.75 (*s*, 3H, 4′-$OCH_3$), 3.83 (*s*, 3H, 3′-$OCH_3$), 6.56 (*s*, 1H, H-8), 6.94 (*m*, 2H, H-6/H-5′), 7.97 (*d*, 1H, 8.5 Hz, H-6′), 7.75 (*s*, 1H, H-2′), 8.02 (*d*, 1H, 8.4 Hz, H-5), 9,64 (*sl*, 1H, 3-OH), 12.53 (*s*, 1H, 7-OH); $^{13}$C NMR (100 MHz, DMSO- $d_6$) δ 55.8 ($CH_3$, 3′-$OCH_3$), 60.0 ($CH_3$, 4′-$OCH_3$), 93.9 (CH, C-8), 103.4 (C, C-10), 111.8 (C, C-2′), 115.6 (CH, C-6), 121.8 (C, C-1′), 122.0 (CH, C-6′), 130.9 (CH, C-5), 135.5 (C, C-3), 146.8 (C, C-2), 147.4 (C, C-3′), 148.9 (C, C-4′), 151.7 (C, C-9), 157.2 (C, C-7), 176.1 (C, C-4).

**Figure S6.** $^1$H NMR spectrum (400 MHz, DMSO-$d_6$) of compound **2**.

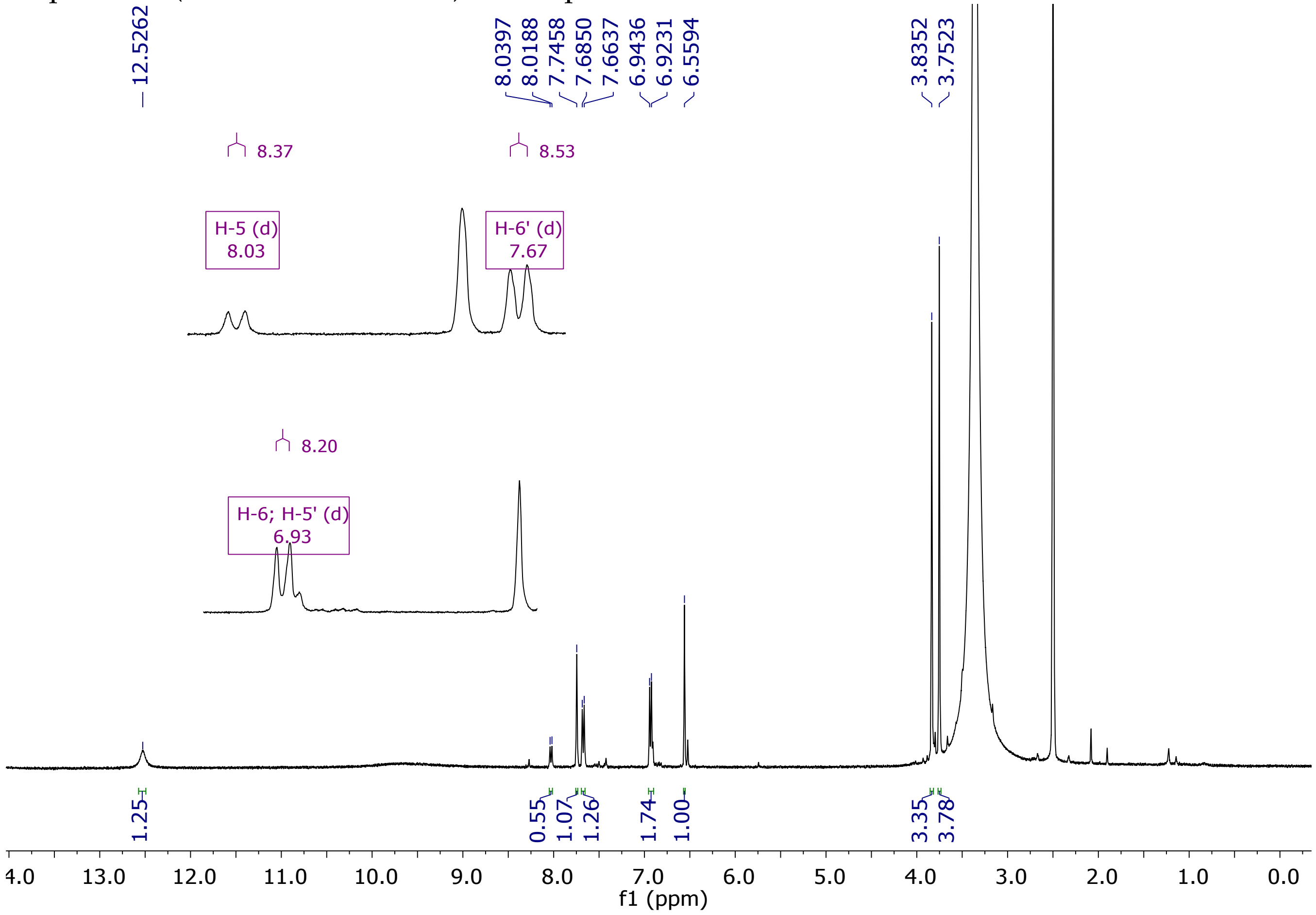

**Figure S7.** $^{13}$C NMR spectrum (100 MHz, DMSO-$d_6$) of compound **2**.

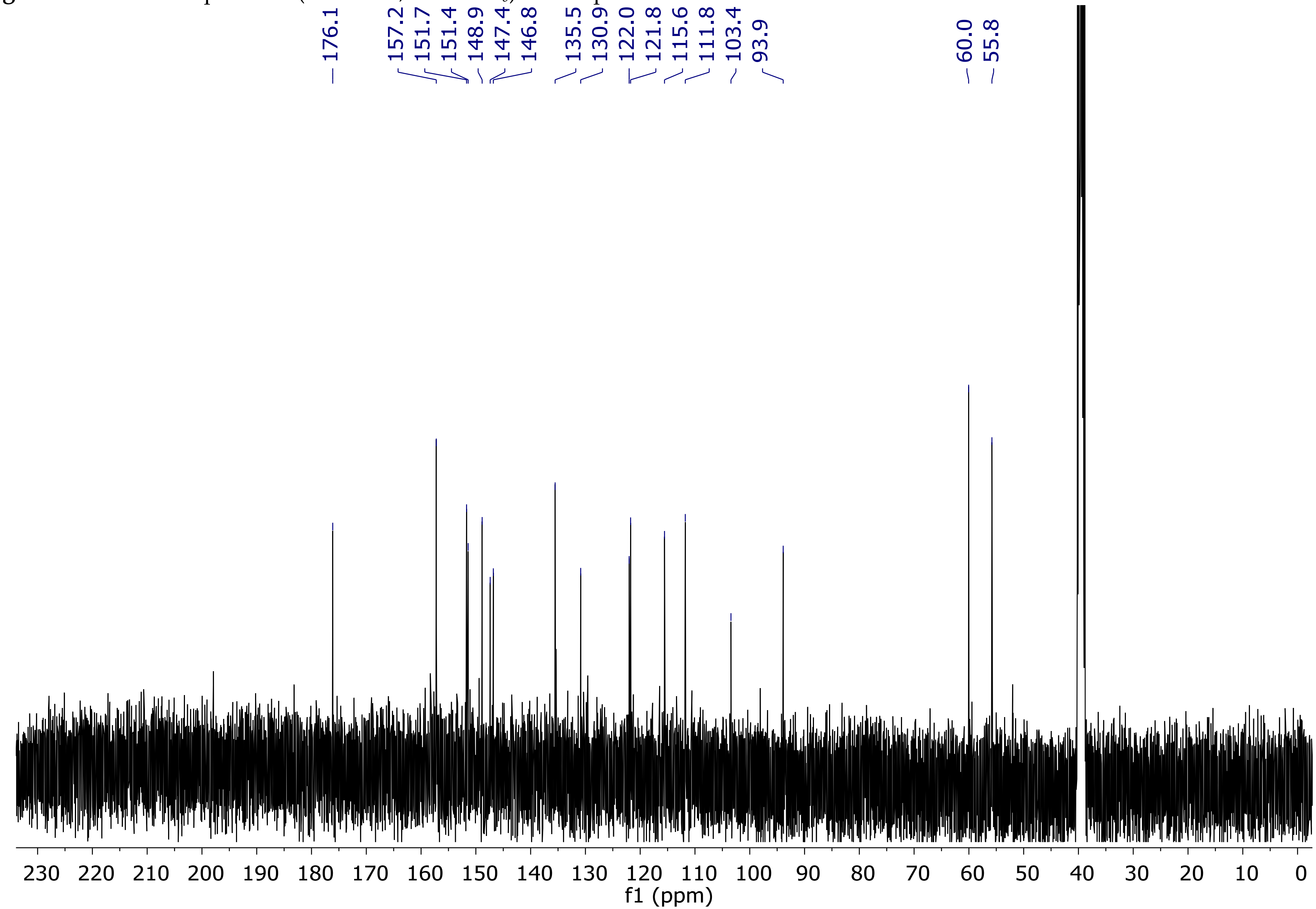

**Figure S8.** $^{1}H$ x $^{1}H$ (COSY) NMR spectrum (400 MHz, DMSO-$d_6$) of compound **2**.

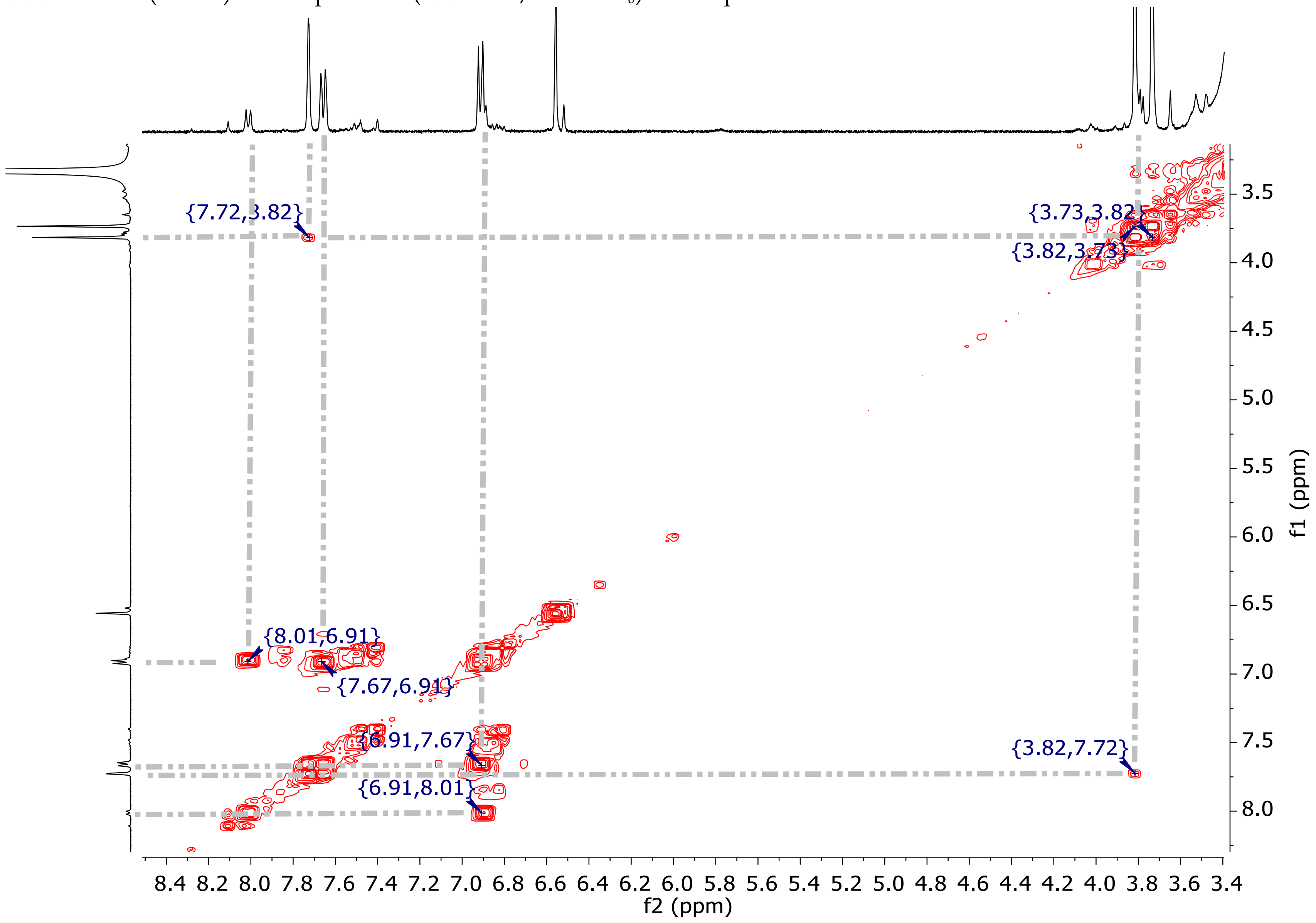

**Figure S9.** $^{1}H$ x $^{13}C$ (HSQC) NMR spectrum of compound **2**.

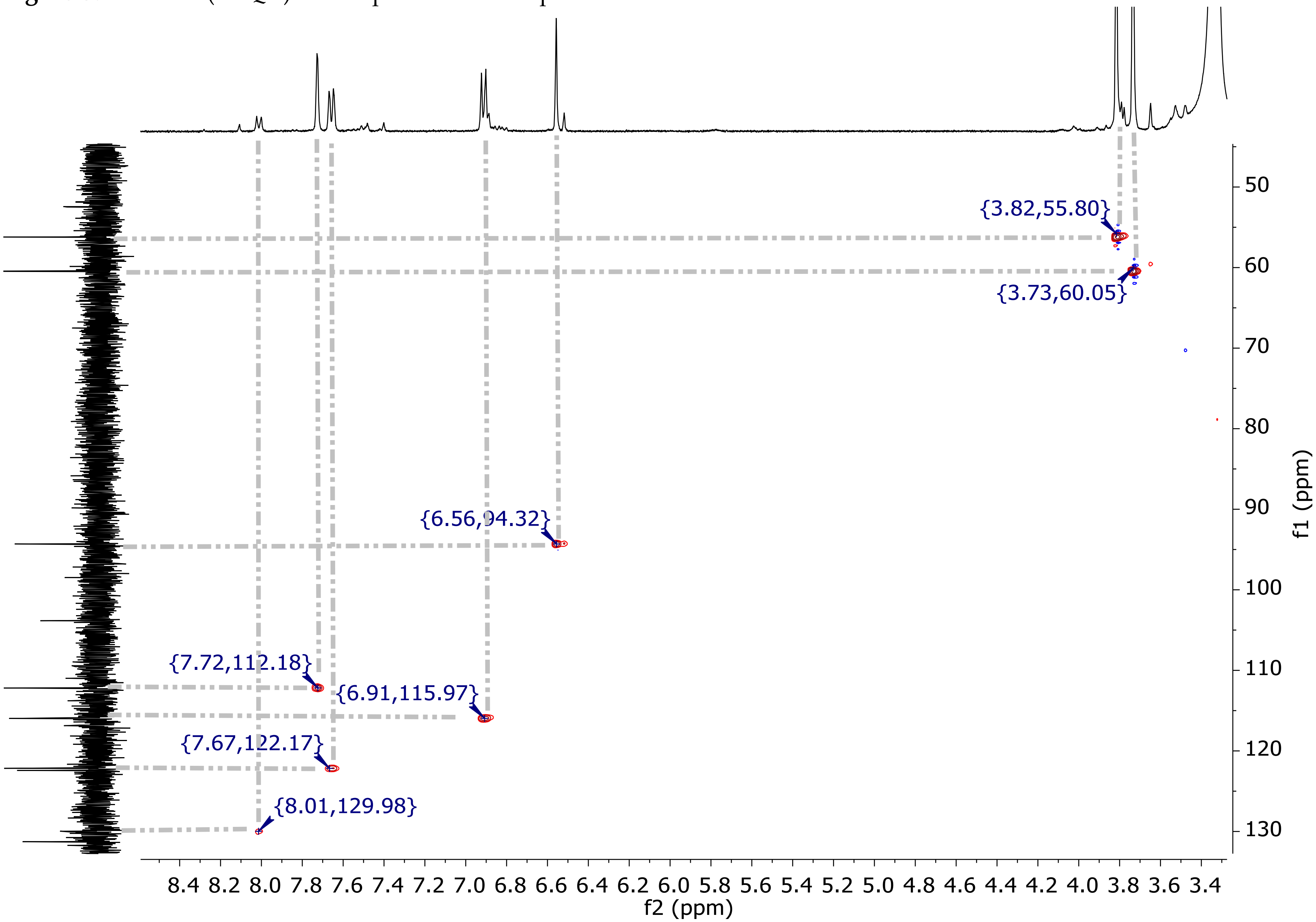

**Figure S10.** $^1H$ x $^{13}C$ (HMBC) NMR spectrum of compound **2**.

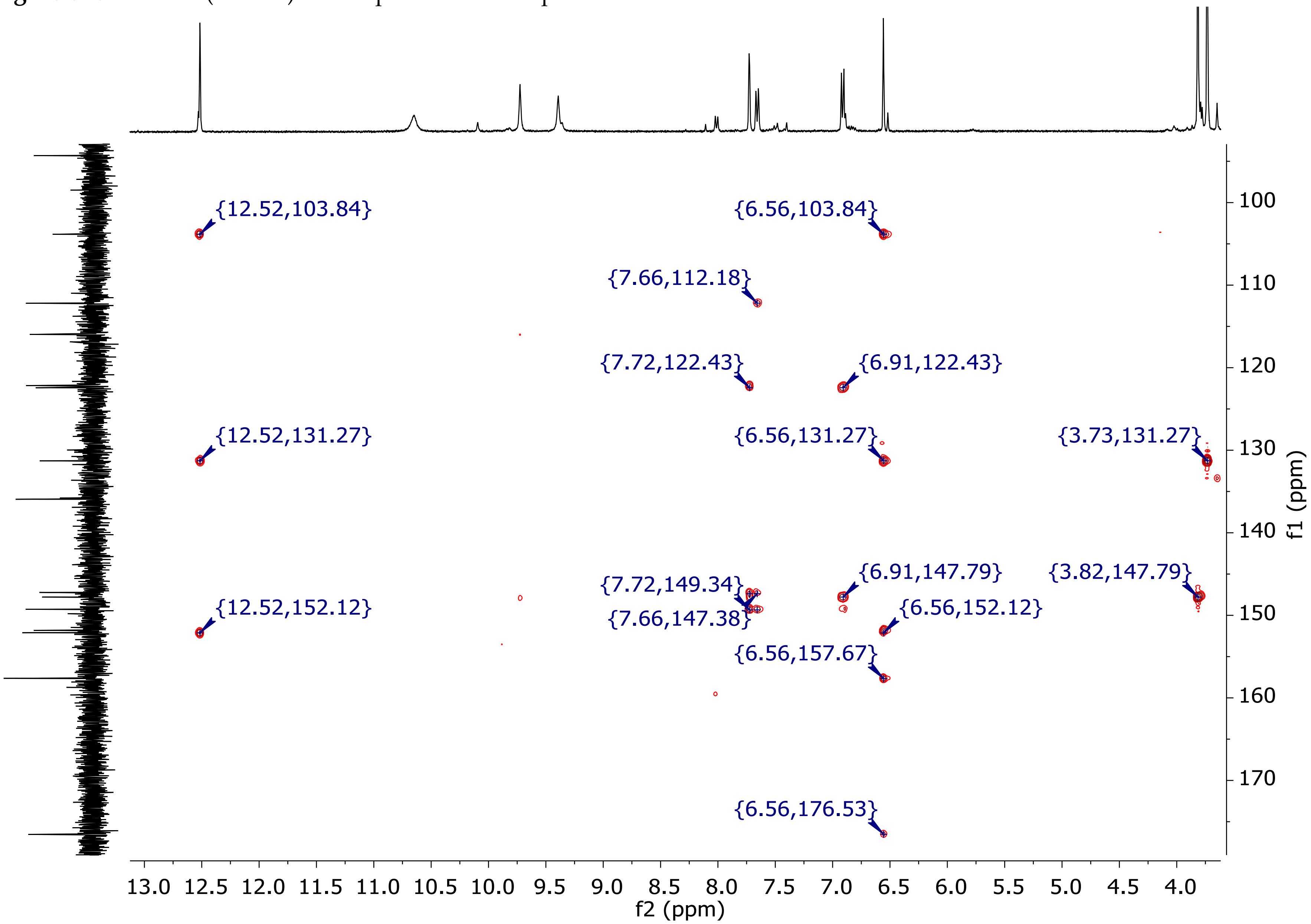

*p*-hydroxybenzoic acid (**3**): $^1$H NMR (400 MHz, DMSO-$d_6$) δ 6.71 (*d*, 2H, 8.0 Hz, H-3/H-5), 7.71 (*d*, 2H, 8.0 Hz, H-2/H-6); $^{13}$C NMR (100 MHz, DMSO-$d_6$)) δ 114.8 (CH, C-4/C-6), 124.2 (C, C-2), 131.4 (CH, C-3/C-7), 160.9 (C, C-5), 171,9 (C, C-1).

**Figure S11**. $^1$H NMR spectrum (400 MHz, DMSO-$d_6$) of compound **3**.

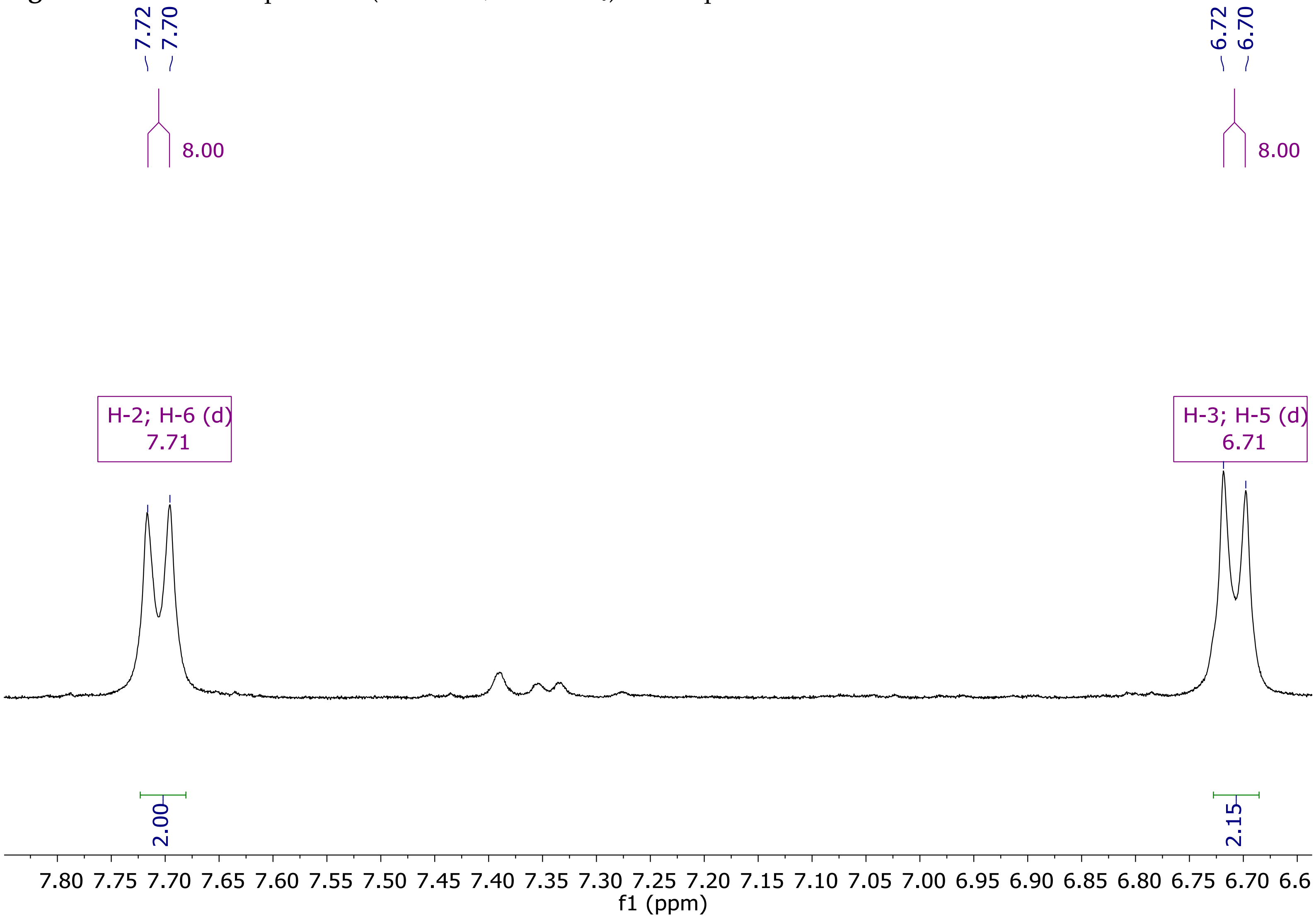

**Figure S12**. $^{1}H$ x $^{13}C$ (HSQC) NMR spectrum of compound **3**.

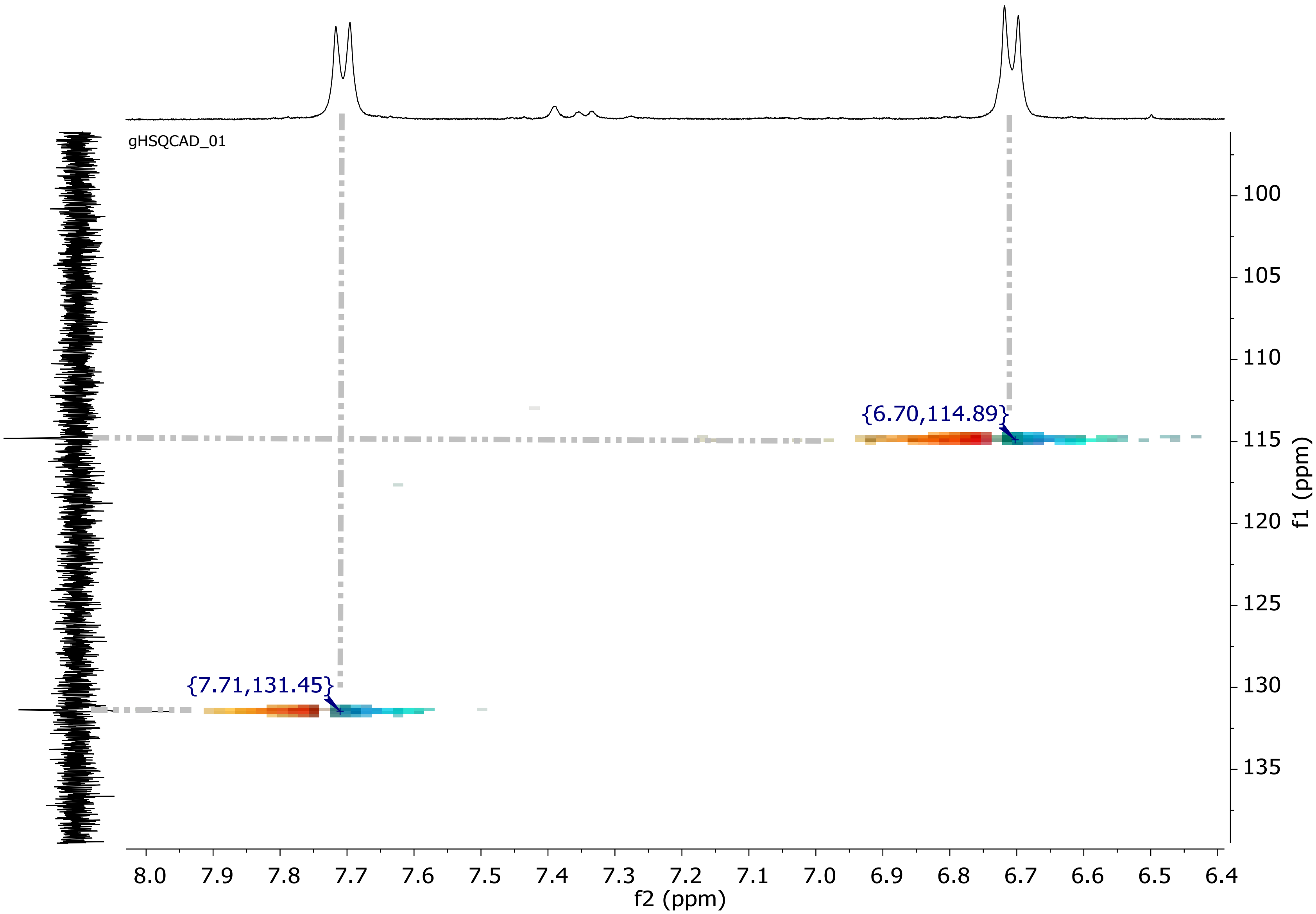

**Figure S13**. $^{1}H$ x $^{13}C$ (HMBC) NMR spectrum of compound **3**.

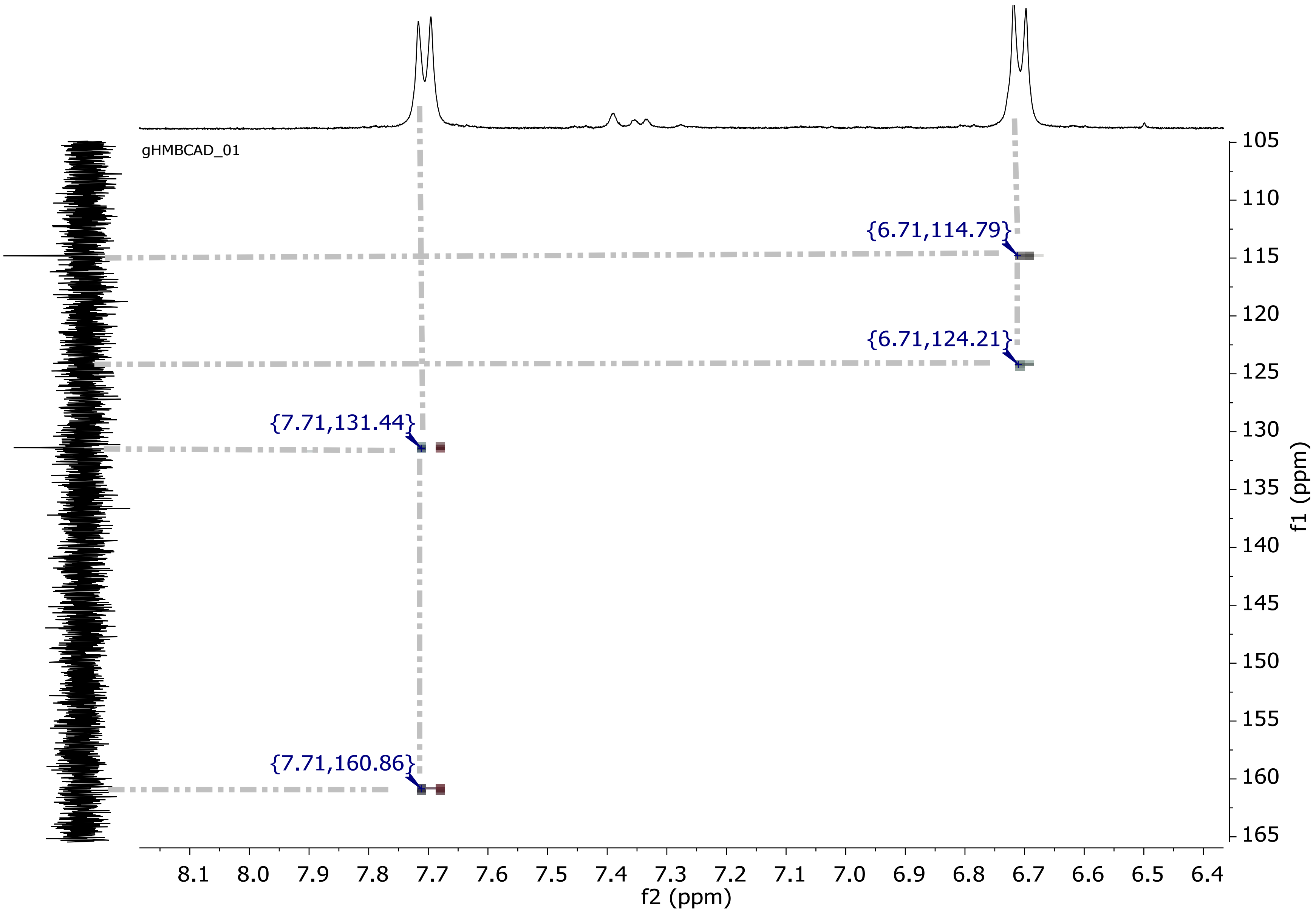

6-methoxykaempferol-3-*O*-β-D-6′′-(*p*-coumaroyl)-glycopyranoside (**4**): $^{1}$H NMR (400 MHz, $CD_3OD$) *δ* 3.42-3.40 (*m*, 4H, H-2″/ H-3″/ H-4″/ H-5″), 3.83 (*s*, 3H, 6-$OCH_3$), 4.19 (*m*, 1H, 6b″), 4.30 (*dd*, 1H, 12.0 and 4.0 Hz, 6a″), 5.24 (*d*, 1H, 8.0 Hz, H-1″), 6.08 (*d*, 2H, 16.0 Hz, H-a), 6.43 (*s*, 1H, H-8), 6.80 (*d*, 2H, 8.0 Hz, H-3′″/H-5′″), 6.82 (*d*, 2H, 8.0 Hz, H-3′/H-5′), 7.31 (*d*, 2H, 8.0 Hz, H-2′″/H-6′″), 7.40 (*d*, 2H, 16.0 Hz, H-b), 8.00 (*d*, 2H, 8.0 Hz, H-2′/H-6′); $^{13}$C NMR (100 MHz, $CD_3OD$) *δ* 60.7 ($CH_3$, 6-$OCH_3$), 64.2 ($CH_2$, C-6a′′/C-6b′′), 71.6 (CH, C-4′′), 75.7 (CH, C-5′′), 75.8 (CH, C-2′′), 77.7 (CH, C-3′′), 95.0 (CH, C-8), 103.7 (CH, C-1′′), 105.9 (C, C-10), 114.7 (CH, C-8′′′), 116.1 (CH, C-3′/C-5′), 116.7 (CH, C-3′′′/C-5′′′), 122.7 (C, C-1′), 126.9 (C, C-1′′′), 131.1 (CH, C-2′′′/C-6′′′), 132.2 (CH, C-2′/C-6′), 132.6 (C, C-6), 134.8 (C, C-3), 146.4 (CH, C-7′′′), 153.6 (C, C-5), 153.7 (C, C-9), 158.6 (C, C-2), 159.4 (C, C-7), 160.9 (C, C-4′′′), 161.6 (C, C-4′), 168.7 (C, C-9′′′), 179.8 (C, C-4); HRMS *m*/*z* 623.14141 (calcd for $C_{31}H_{28}O_{14}$).

**Figure S14**. High-resolution mass spectrum of compound **4** (Negative Mode, Full Scan).

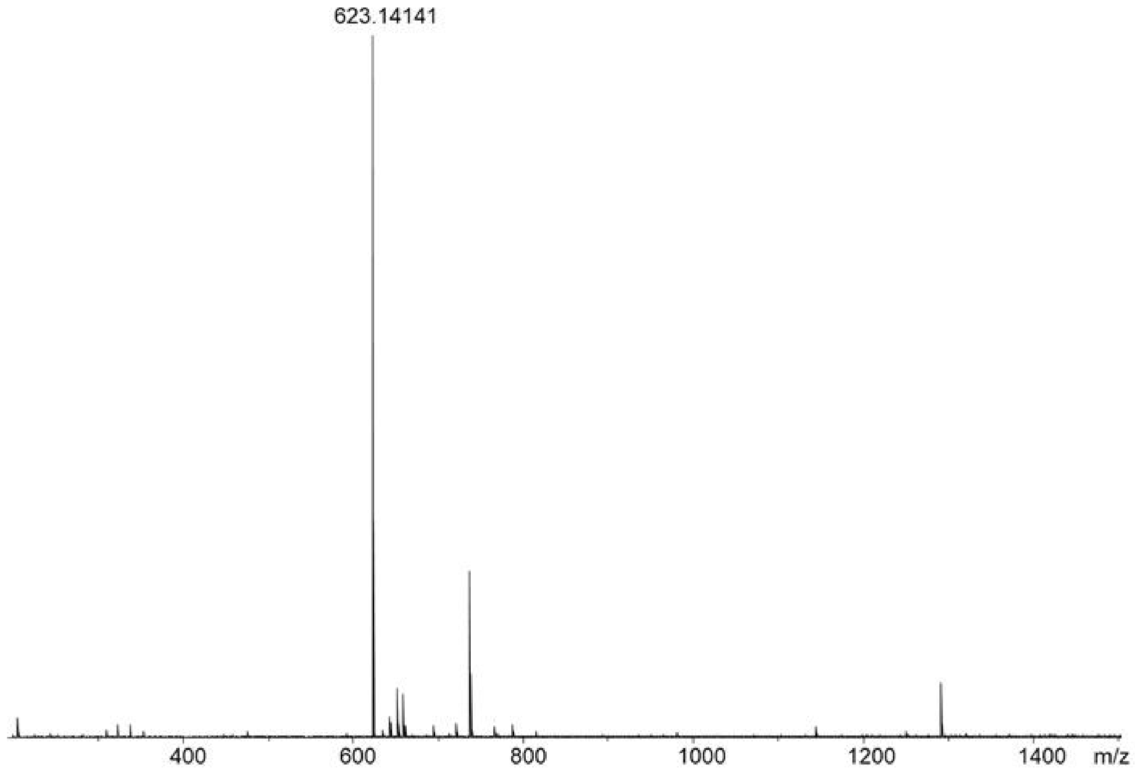

**Figure S15**. $^{1}$H NMR spectrum (400 MHz, $CD_3OD$) of compound **4.**

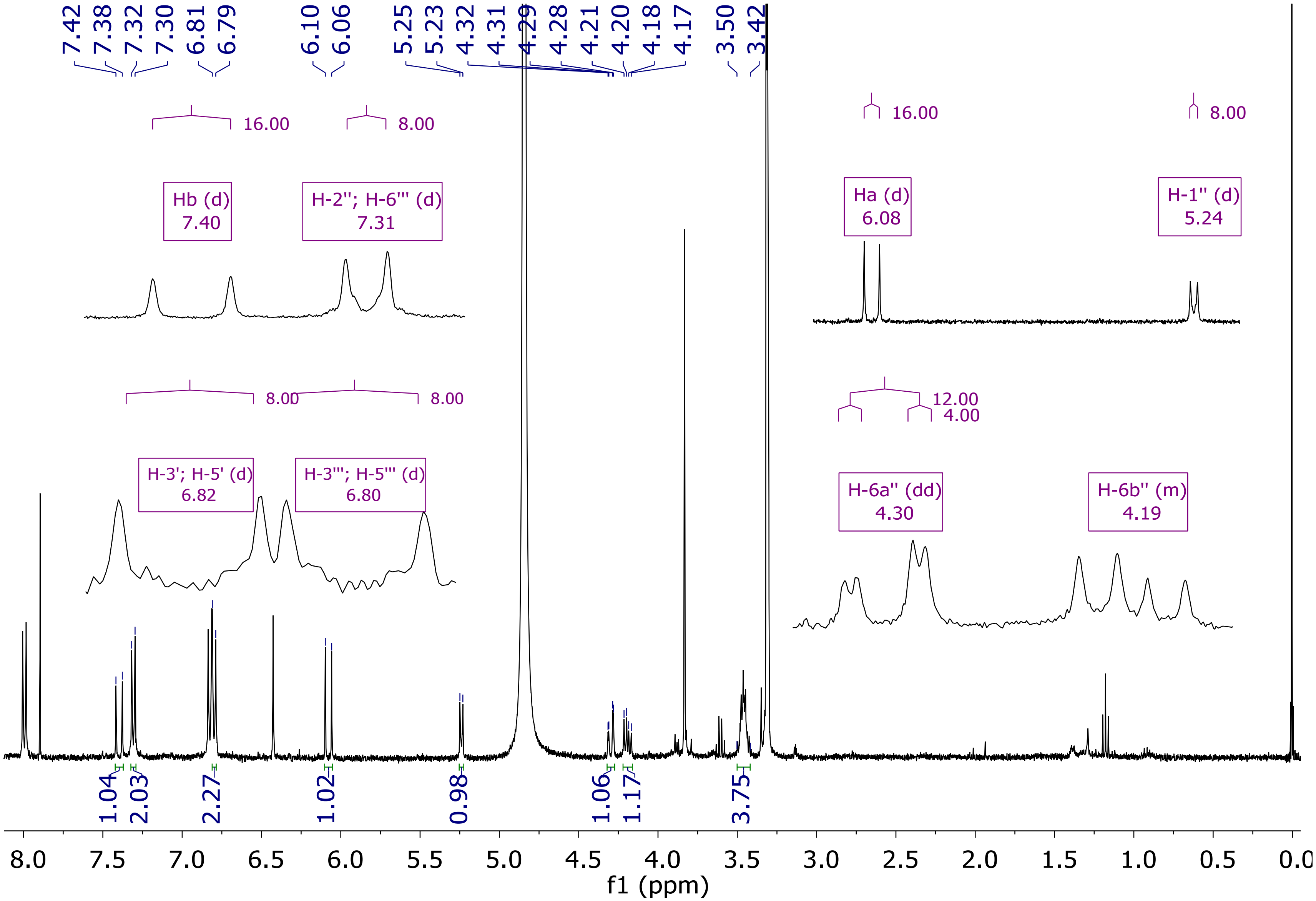

**Figure S16.** $^{13}C$ NMR spectrum (100 MHz, $CD_3OD$) of compound **4**.

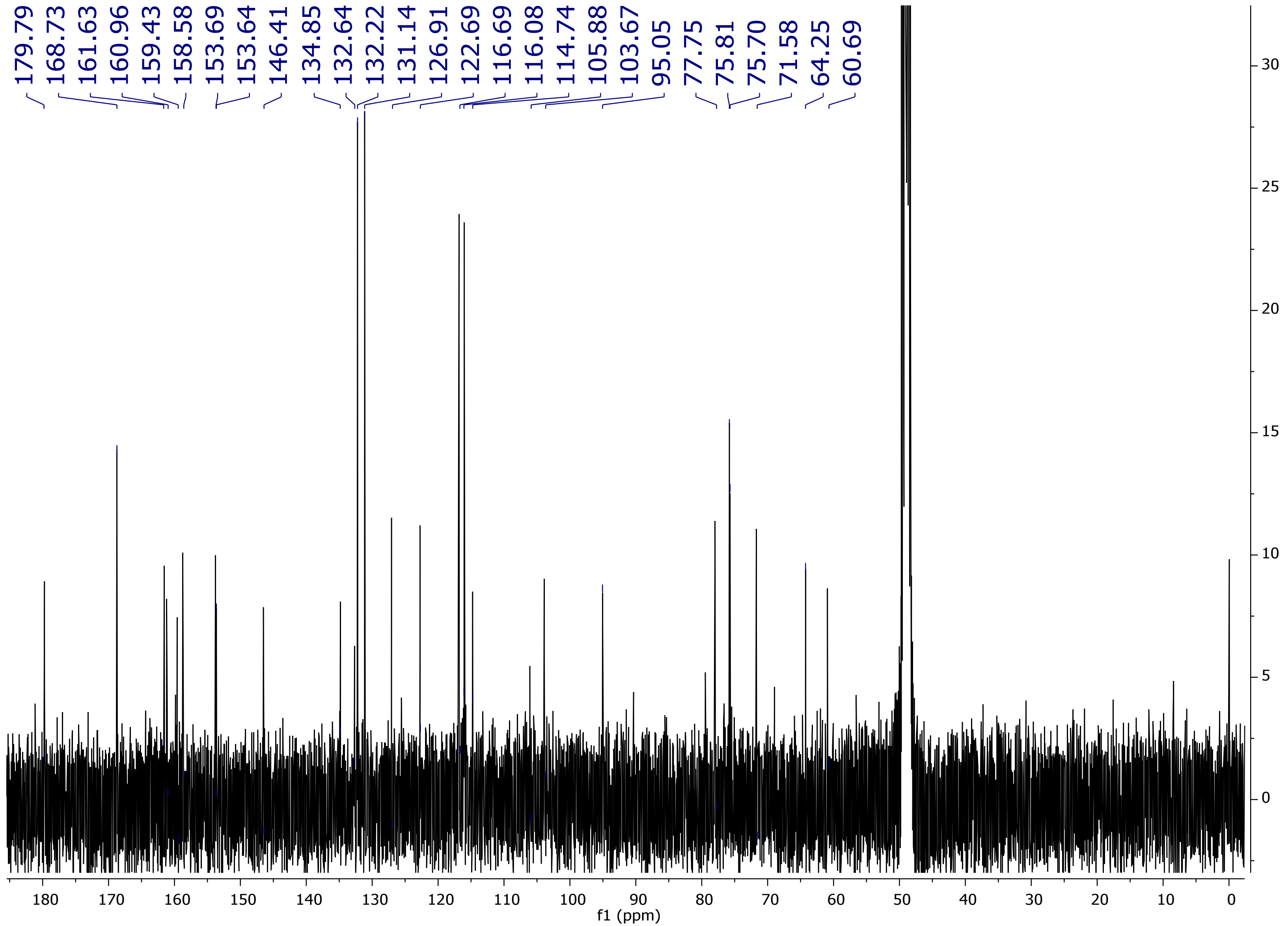

**Figure S17**. $^1H$ x $^1H$ (COSY) NMR spectrum (400 MHz, $CD_3OD$) of compound **4.**

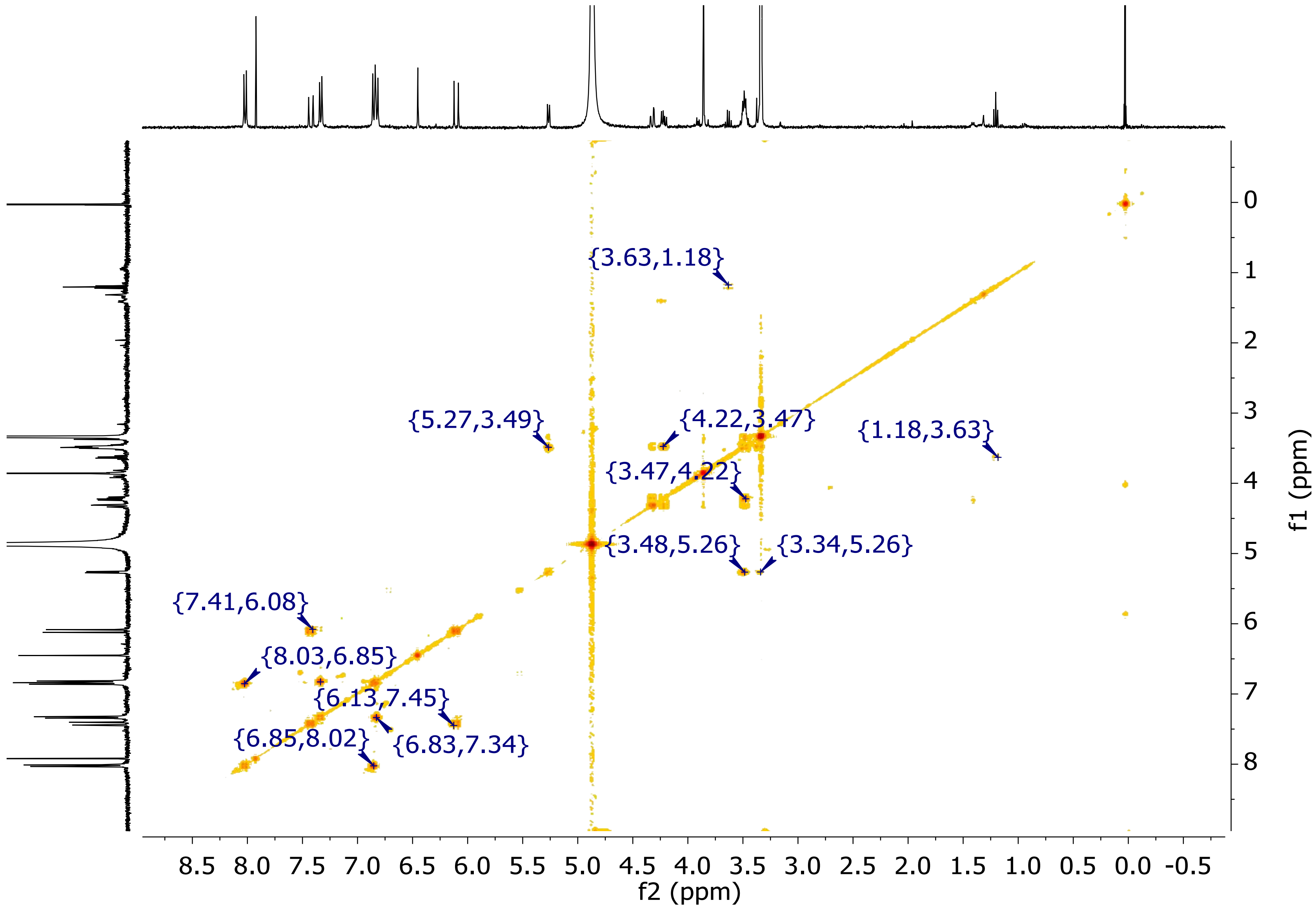

**Figure S18**. $^{1}H$ x $^{13}C$ (HSQC) NMR spectrum of compound **4**.

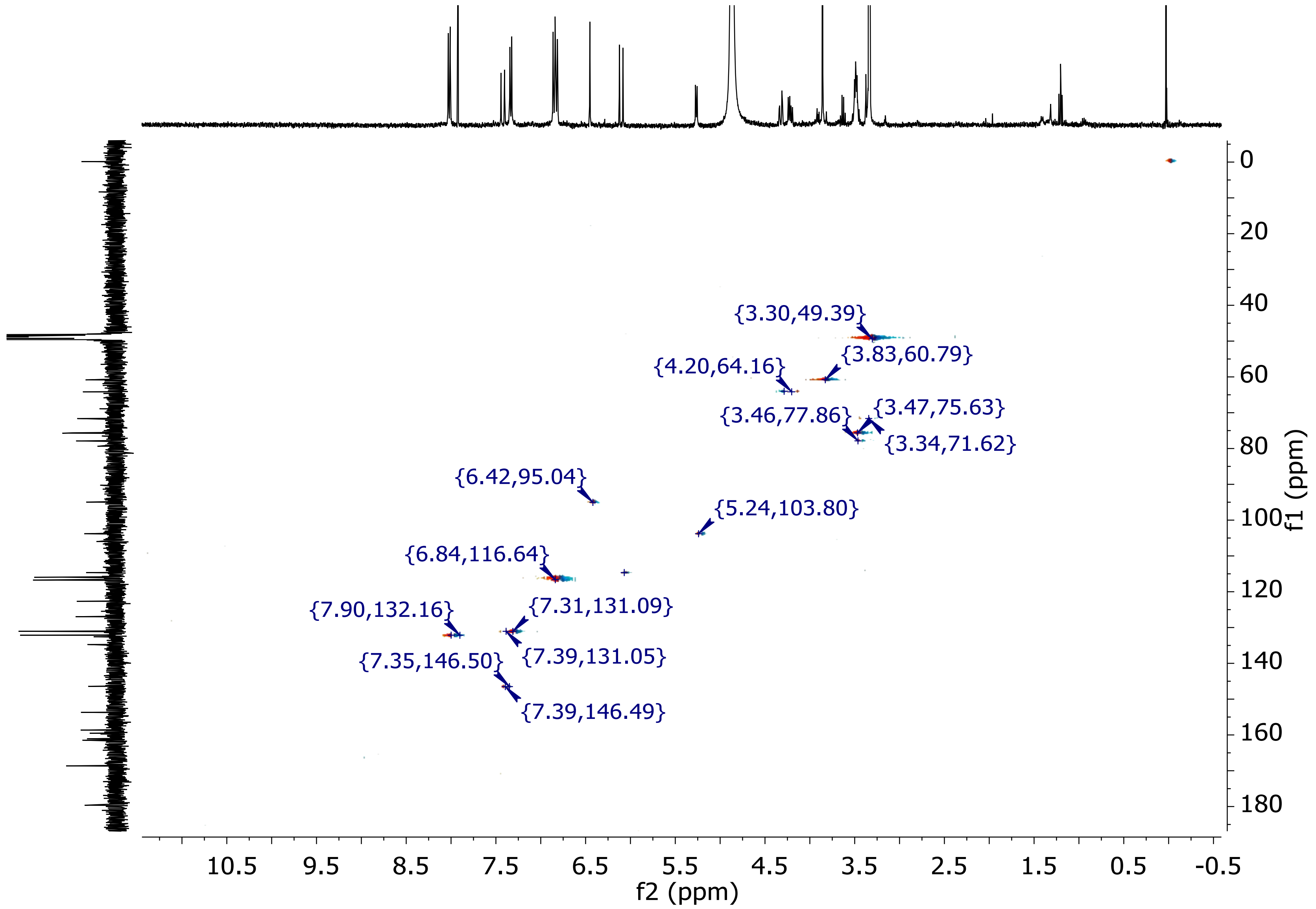

**Figure S19**. $^1$H x $^{13}$C (HMBC) NMR spectrum of compound **4**.

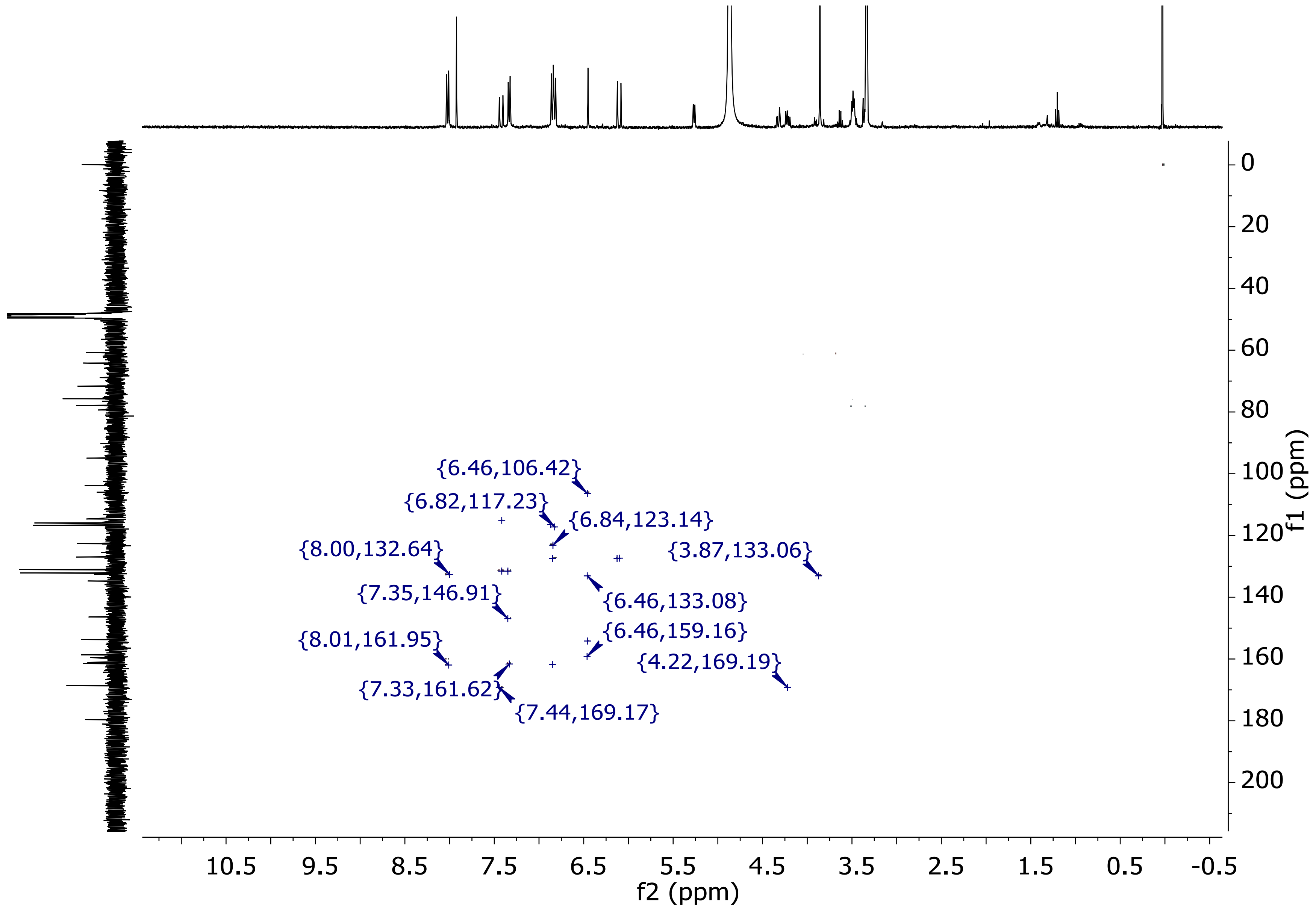

6-methoxykaempferol-3-*O*-β-glucopyranoside (**5**): $^{1}$H NMR (400 MHz, $CD_3OD$) δ 3.44-3.20 (*m*, H-2''/ H-3''/ H-4''/ H-5''), 3.52 (*dd*, 11.9 and 5.5 Hz, 1H, H-6b''), 3.68 (*dd*, 11.9 and 2.4 Hz, 1H, H-6a''), 3.86 (*s*, 3H, 6-$OCH_3$), 5.22 (*d*, 7.3 Hz, 1H, H-1''), 6.46 (*s*, 1H, H-8), 6.87 (*d*, 9.0 Hz, 2H, H-3'/H-5'), 8.03 (*d*, 9.0 Hz, 2H, H-2'/H-6'); $^{13}$C NMR (100 MHz, $CD_3OD$) δ 60.9 ($CH_3$, 6-$OCH_3$), 62.6 ($CH_2$, C-6a''/C-6b''), 71.3 (CH, C-4''), 75.7 (CH, C-2''), 78.0 (CH, C-3''), 78.4 (CH, C-5''), 95.3 (CH, C-8), 104.2 (CH, C-1''), 105.8 (C, C-10), 116.1 (CH, C-3'/C-5'), 122.8 (C, C-1'), 132.3 (CH, C-2'/C-6'), 133.0 (C, C-6), 135.1 (C, C-3), 153.6 (C, C-5), 154.0 (C, C-9), 159.5 (C, C-2), 159.9 (C, C-7), 161.5 (C, C-4'), 179.6 (C, C-4); HRMS *m*/*z* 477.10377 (calcd for $C_{22}H_{22}O_{12}$).

**Figure S20**. High-resolution mass spectrum of compound **5** (Negative Mode, Full Scan).

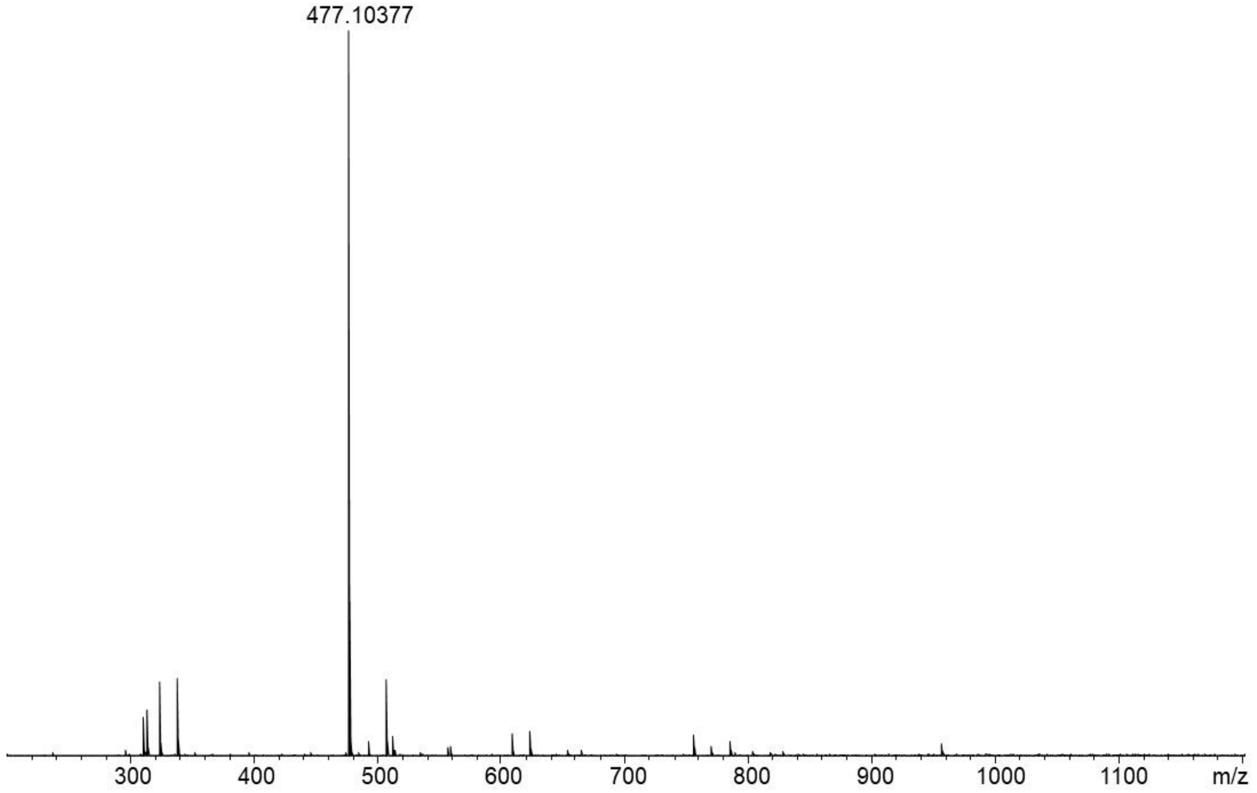

**Figure S21**. $^{1}$H NMR spectrum (400 MHz, $CD_3OD$) of compound **5.**

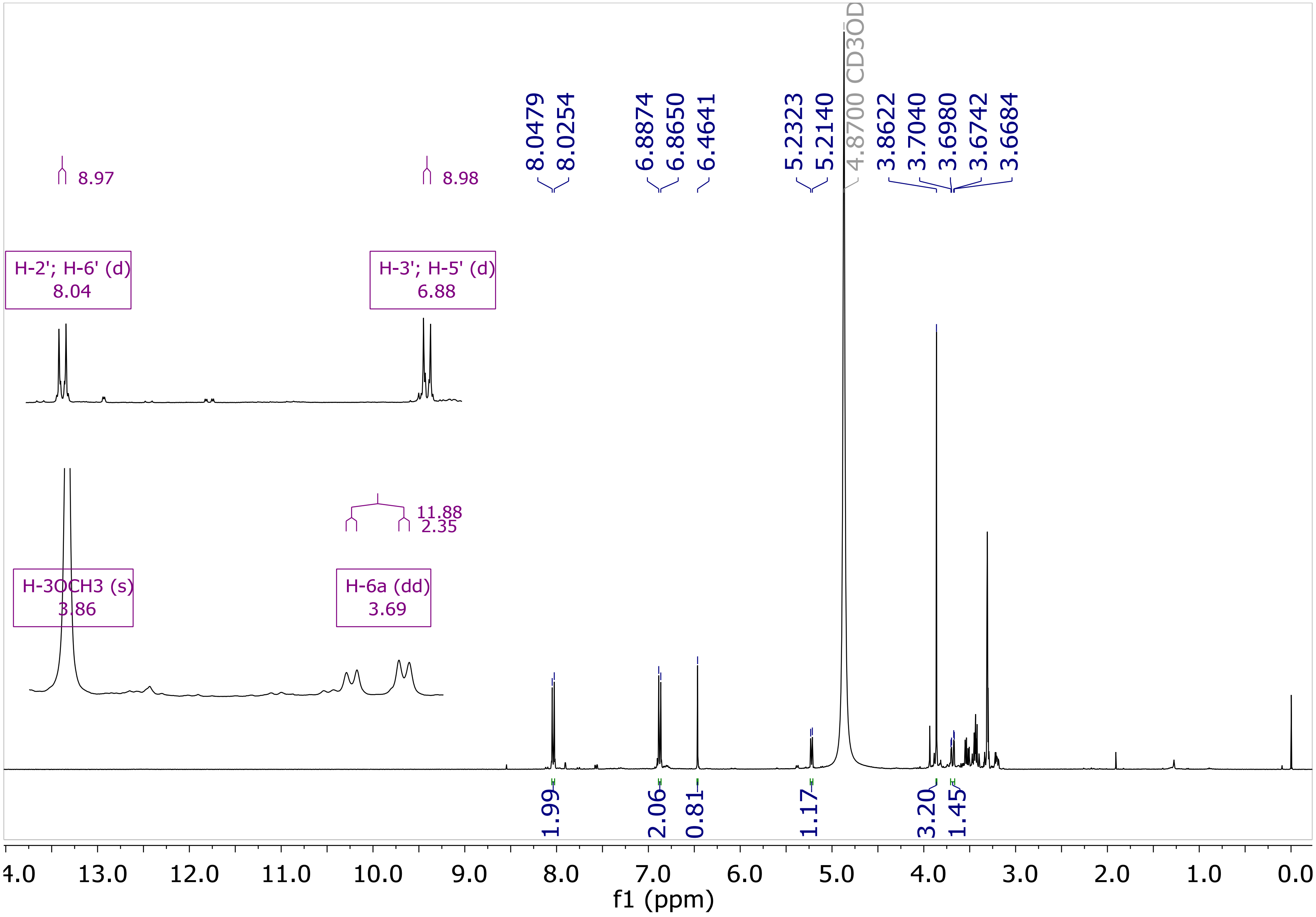

**Figure S22.** $^{13}C$ NMR spectrum (100 MHz, $CD_3OD$) of compound **5**.

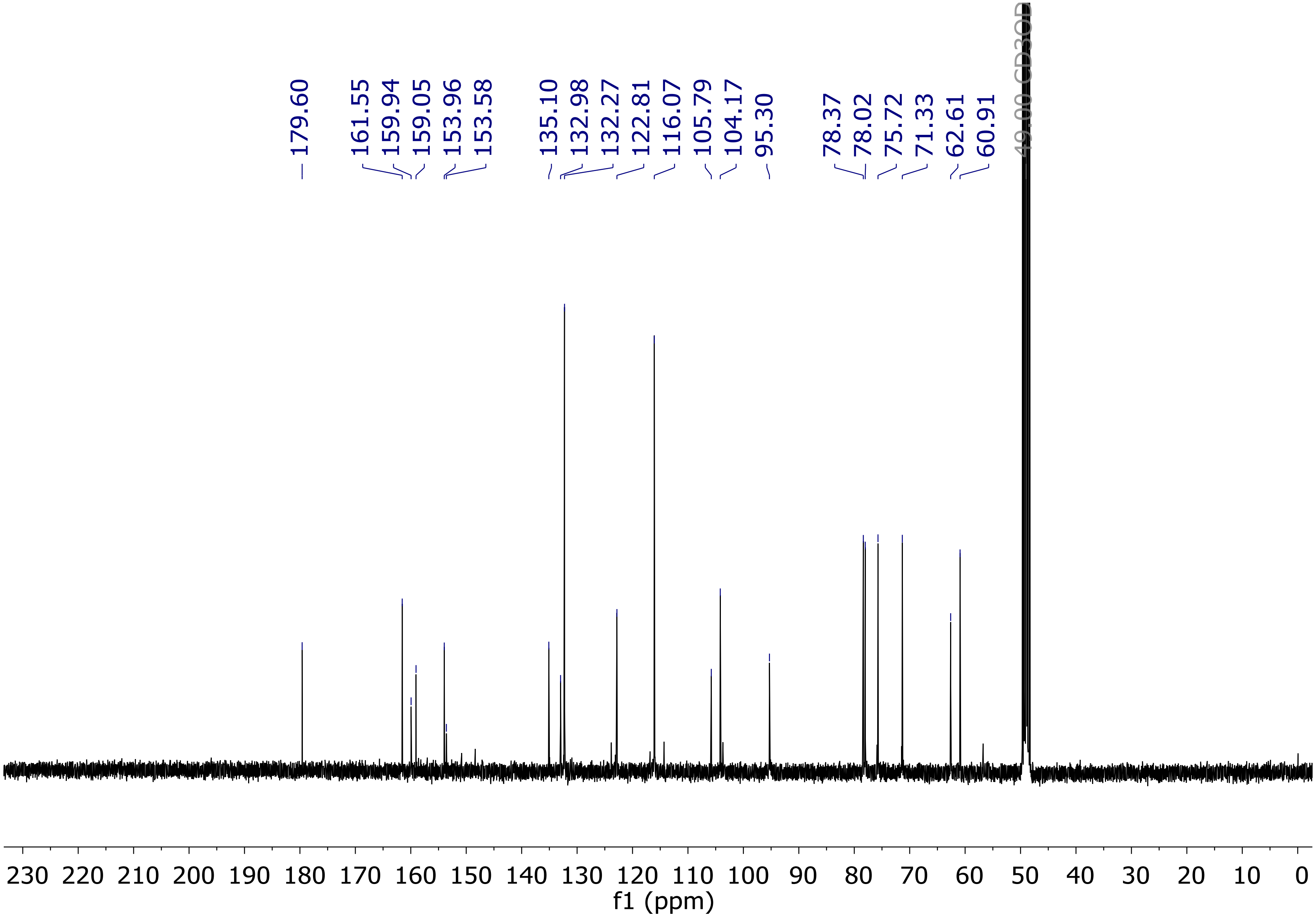

**Figure S23**. $^1H$ x $^1H$ (COSY) NMR spectrum (400 MHz, $CD_3OD$) of compound **5.**

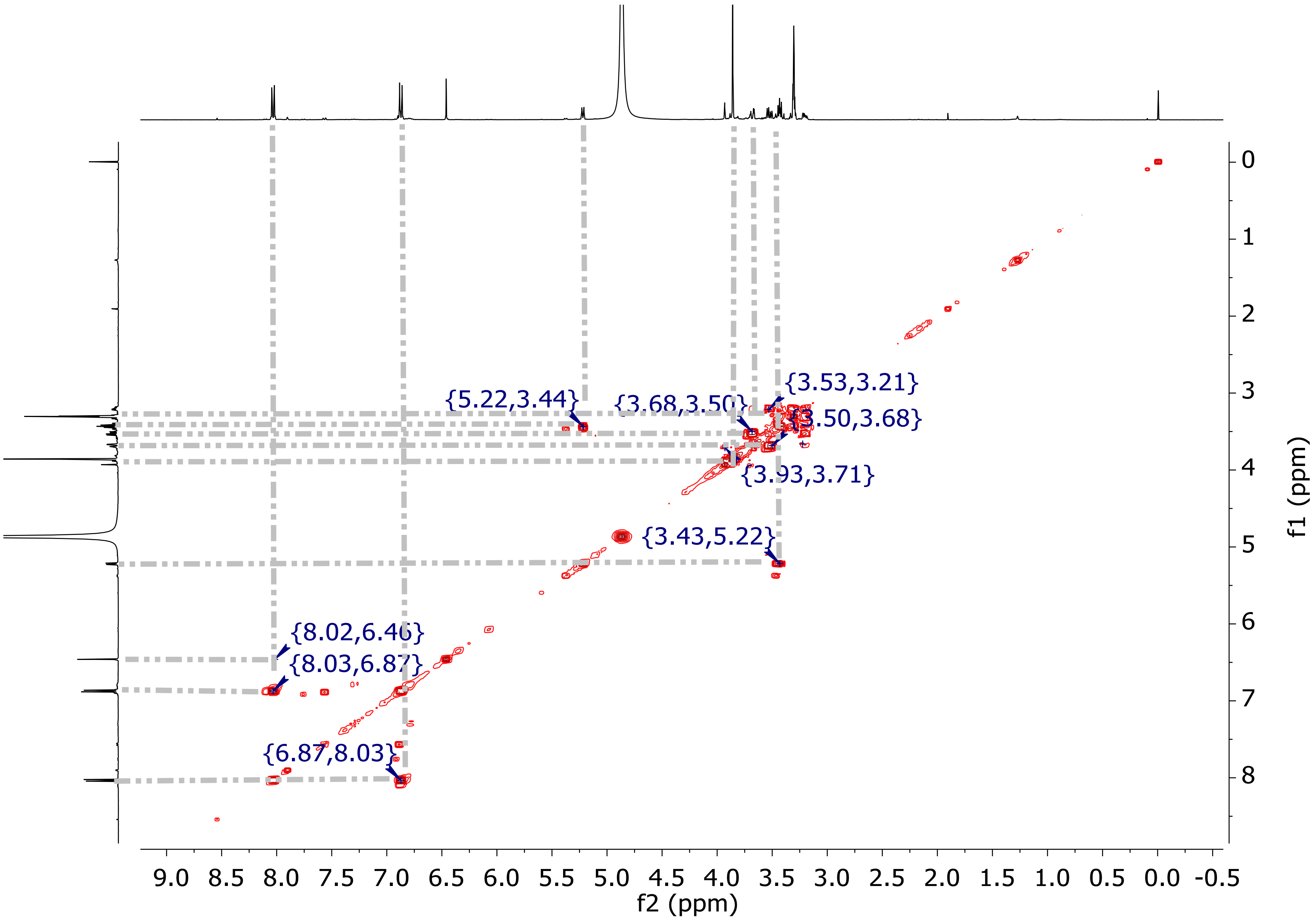

**Figure S24**. $^{1}H$ x $^{13}C$ (HSQC) NMR spectrum of compound **5**.

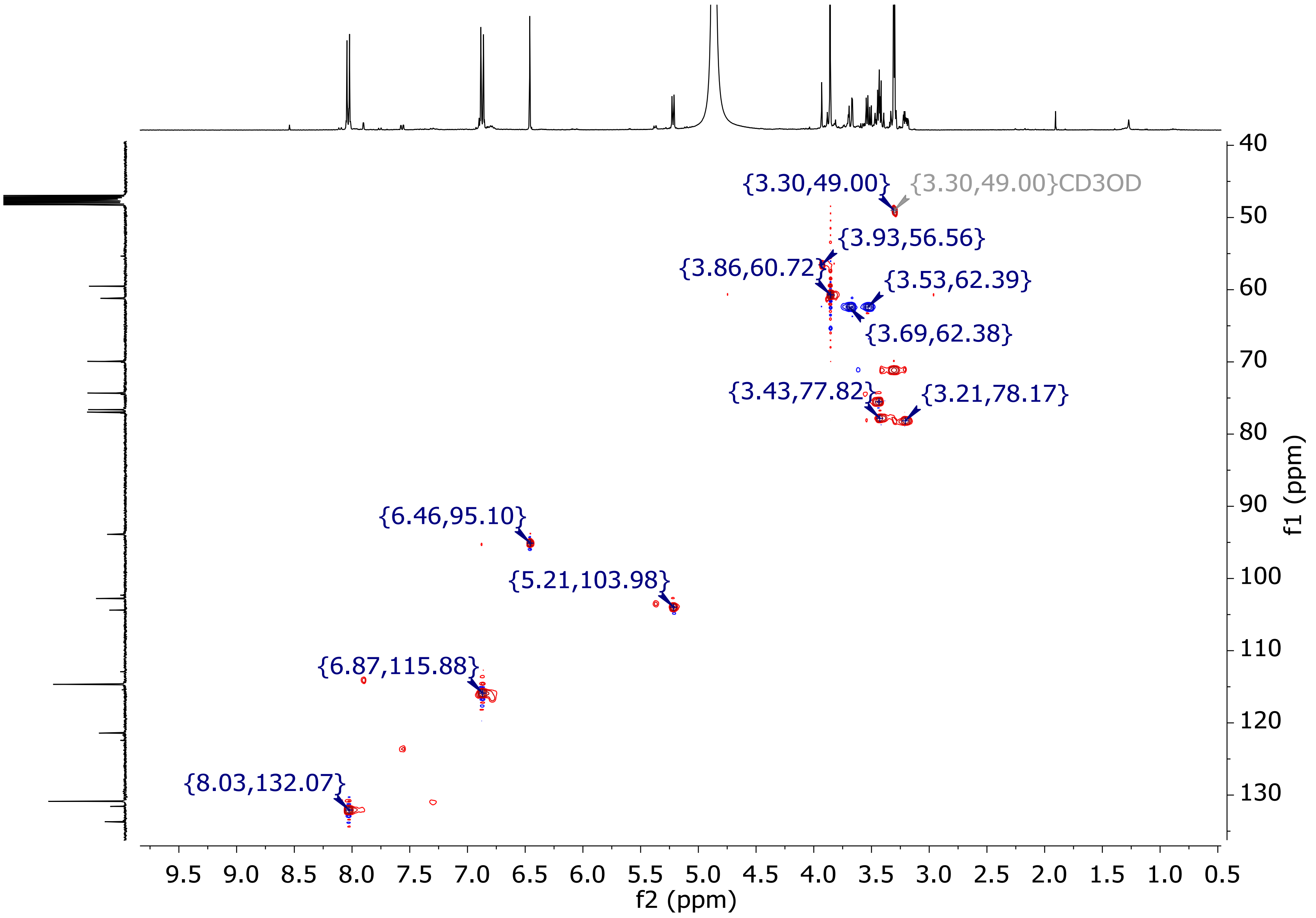

**Figure S25**. $^{1}H$ x $^{13}C$ (HMBC) NMR spectrum of compound **5**.

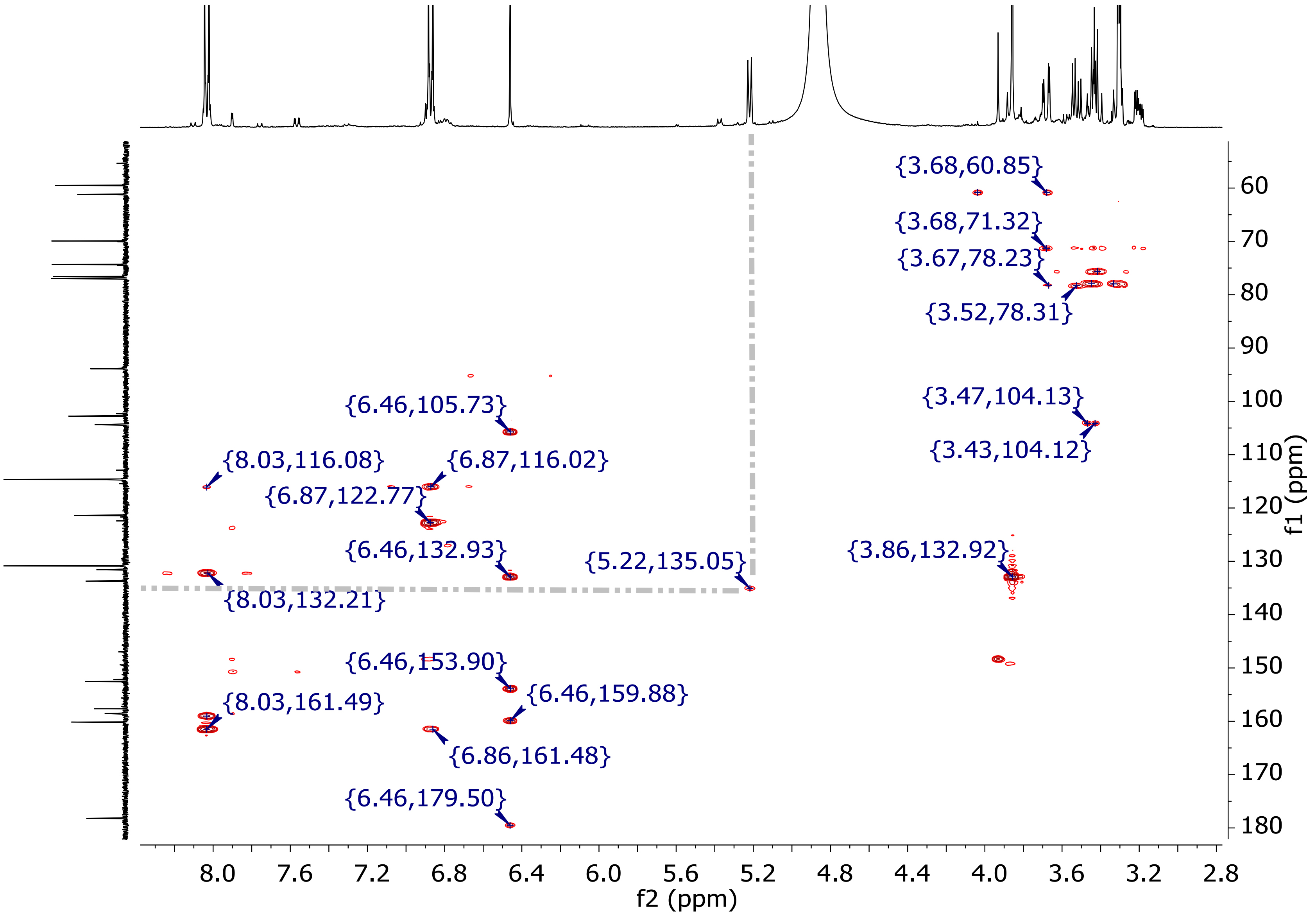

6-methoxykaempferol-7-*O*-β-glucopyranoside (**6**): $^{1}$H NMR (400 MHz, $CD_3OD$) *δ* 3.75-3.40 (*m*, H-2′′/ H-3′′/ H-4′′/ H-5′′), 3.89 (*s*, 3H, 6-$OCH_3$), 3.96 (*dd*, 12.2 and 1.68 Hz, 2H, H-6a′′/H-6b′′), 5.10 (*d*, 7.3 Hz, 1H, H-1′′), 6.88 (*d*, 8.6 Hz, 2H, H-3′/H-5′), 6.91 (*s*, 1H, H-8), 8.15 (*d*, 8.8 Hz, 2H, H-2′/H-6′)′ $^{13}$C NMR (100 MHz, $CD_3OD$) *δ* 61.5 ($CH_3$, 6-$OCH_3$), 62.5 ($CH_2$, C-6a′′/C-6b′′), 71.3 (CH, C-4′′), 74.8 (CH, C-5′′), 77.9 (CH, C-2′′), 78.5 (CH, C-3′′), 95.3 (CH, C-8), 101.9 (CH, C-1′′), 106.7 (C, C-10), 116.5 (CH, C-3′/C-5′), 123.3 (C, C-1′), 130.9 (CH, C-2′/C-6′), 133.3 (C, C-6), 137.6 (C, C-3), 149.3 (C, C-2), 153.1 (C, C-5), 153.2 (C, C-9), 157.5 (C, C-7), 161.3 (C, C-4′), 177.8 (C, C-4); HRMS *m*/*z* 477.1 (calcd for $C_{22}H_{22}O_{12}$).

**Figure S26**. High-resolution mass spectrum of compound **6** (Negative Mode, Full Scan).

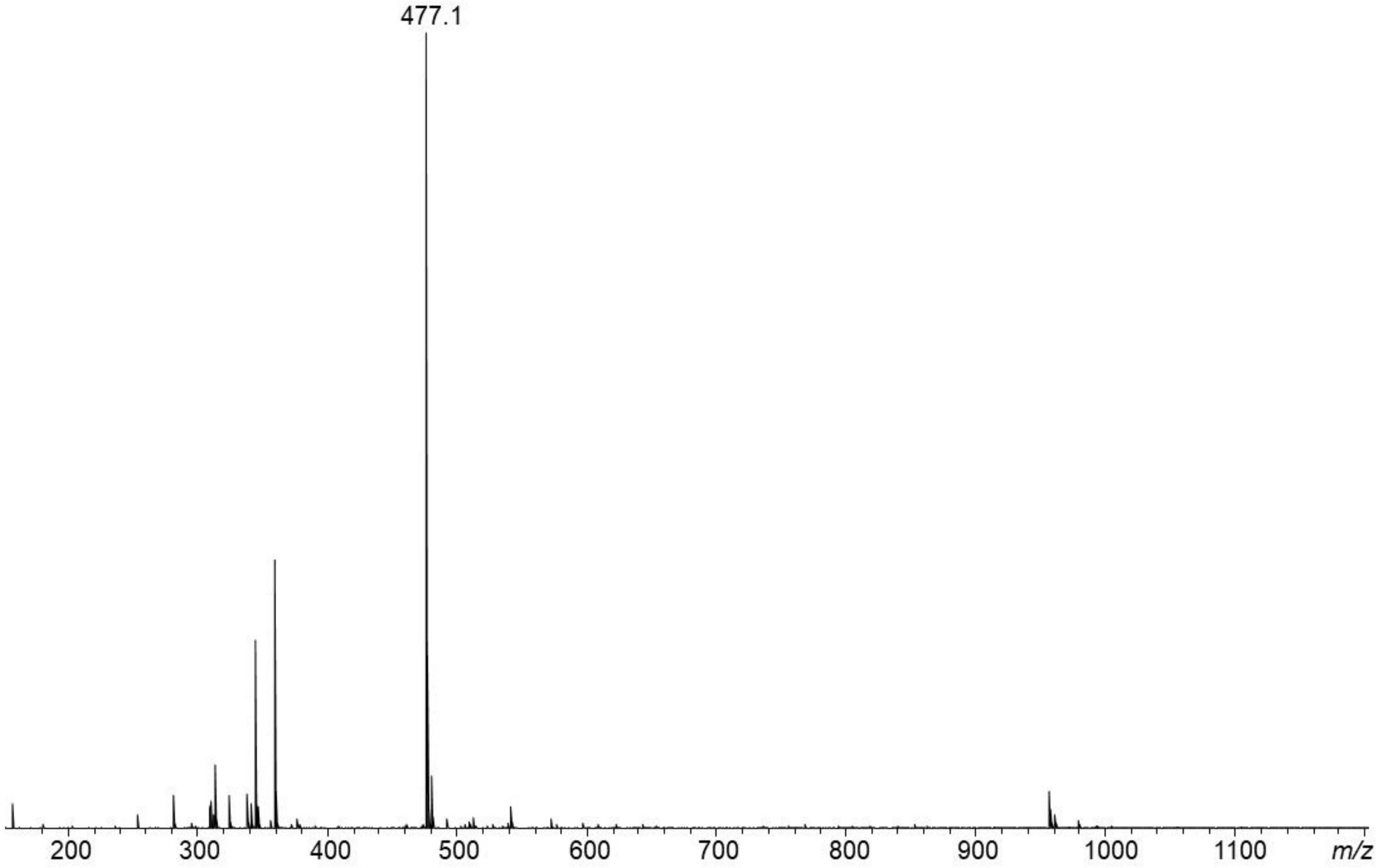

**Figure S27**. $^{1}$H NMR spectrum (400 MHz, $CD_3OD$) of compound **6.**

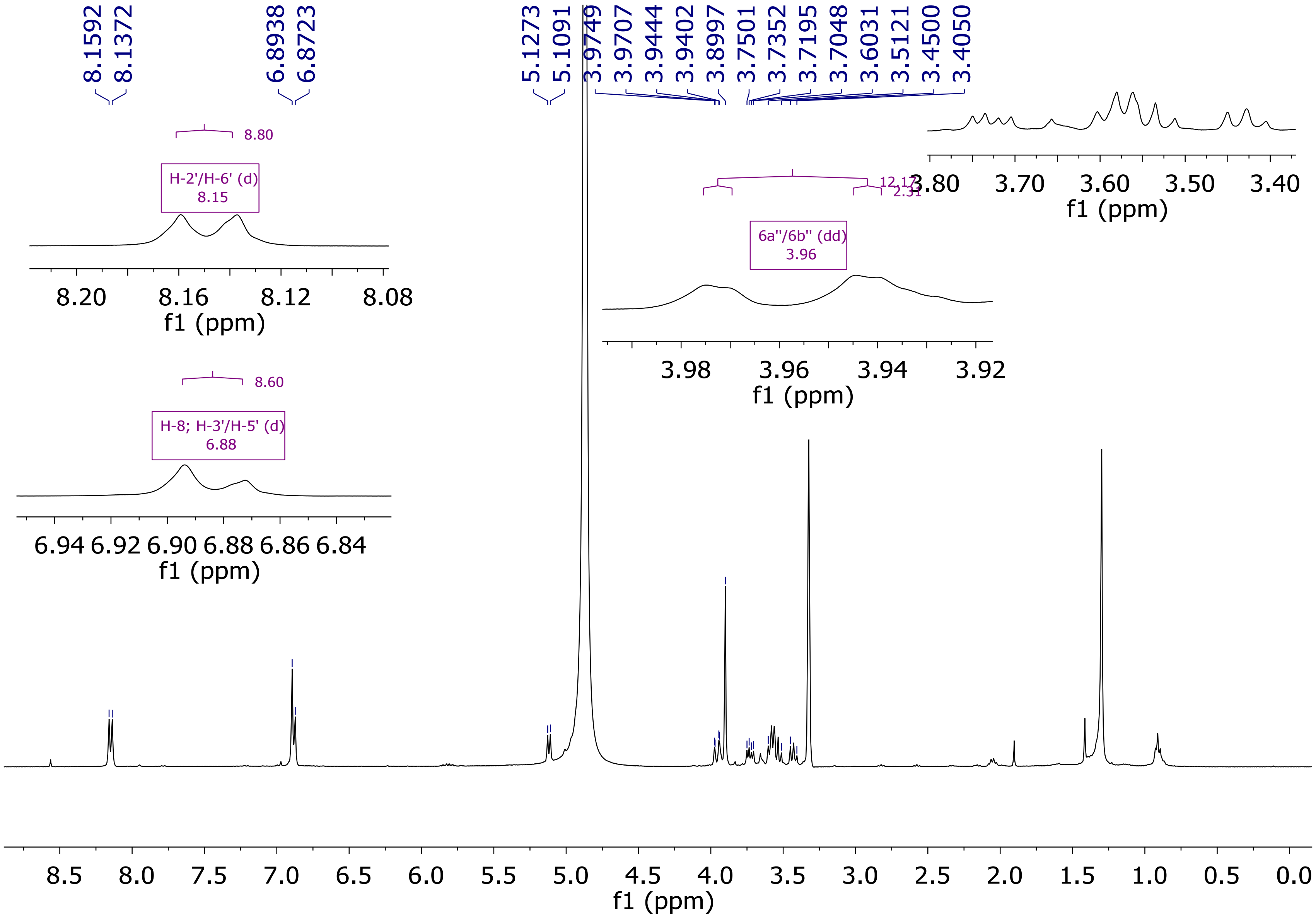

**Figure S28.** $^{13}C$ NMR spectrum (100 MHz, $CD_3OD$) of compound **6**.

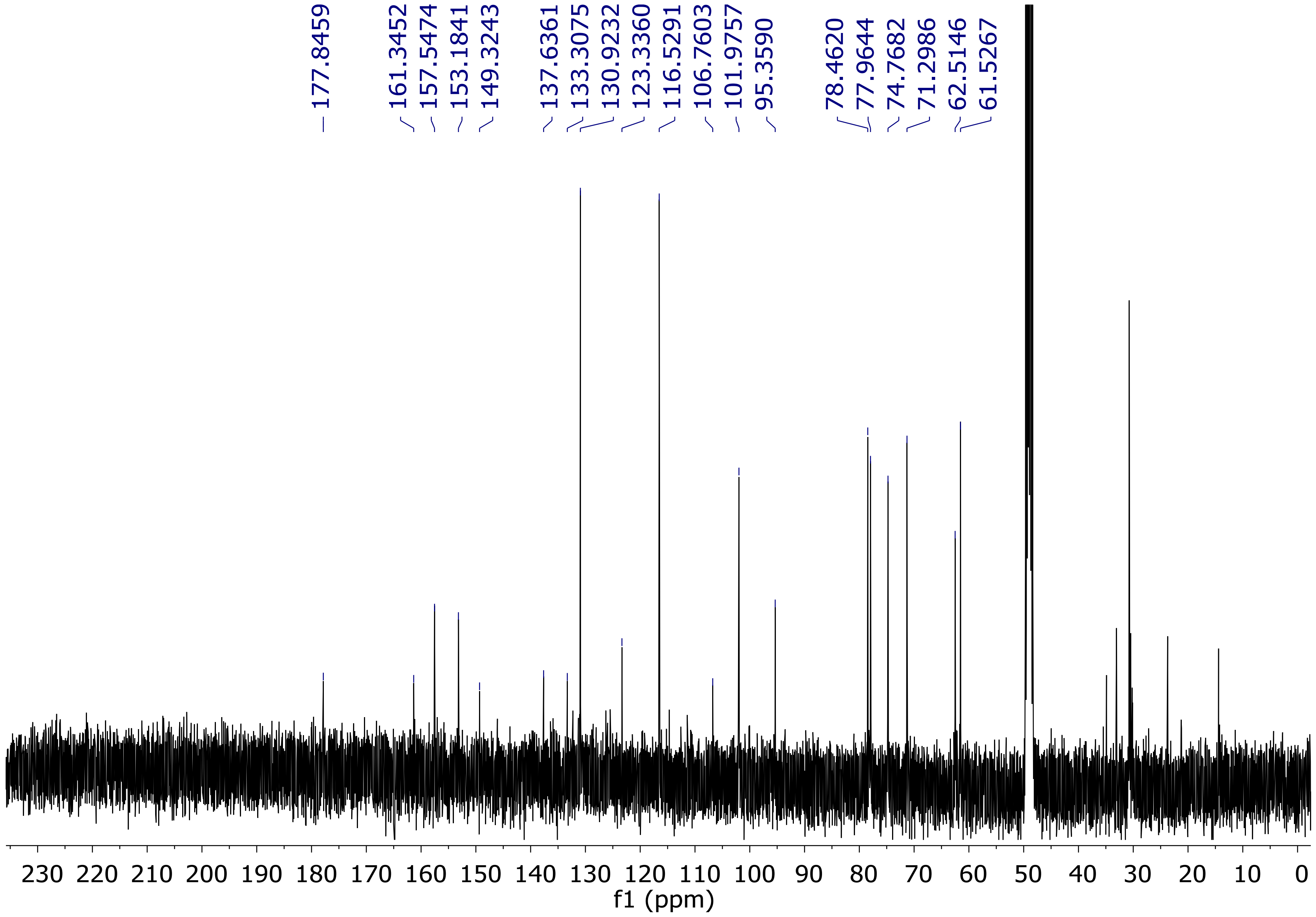

**Figure S29**. $^1H$ x $^1H$ (COSY) NMR spectrum (400 MHz, $CD_3OD$) of compound **6.**

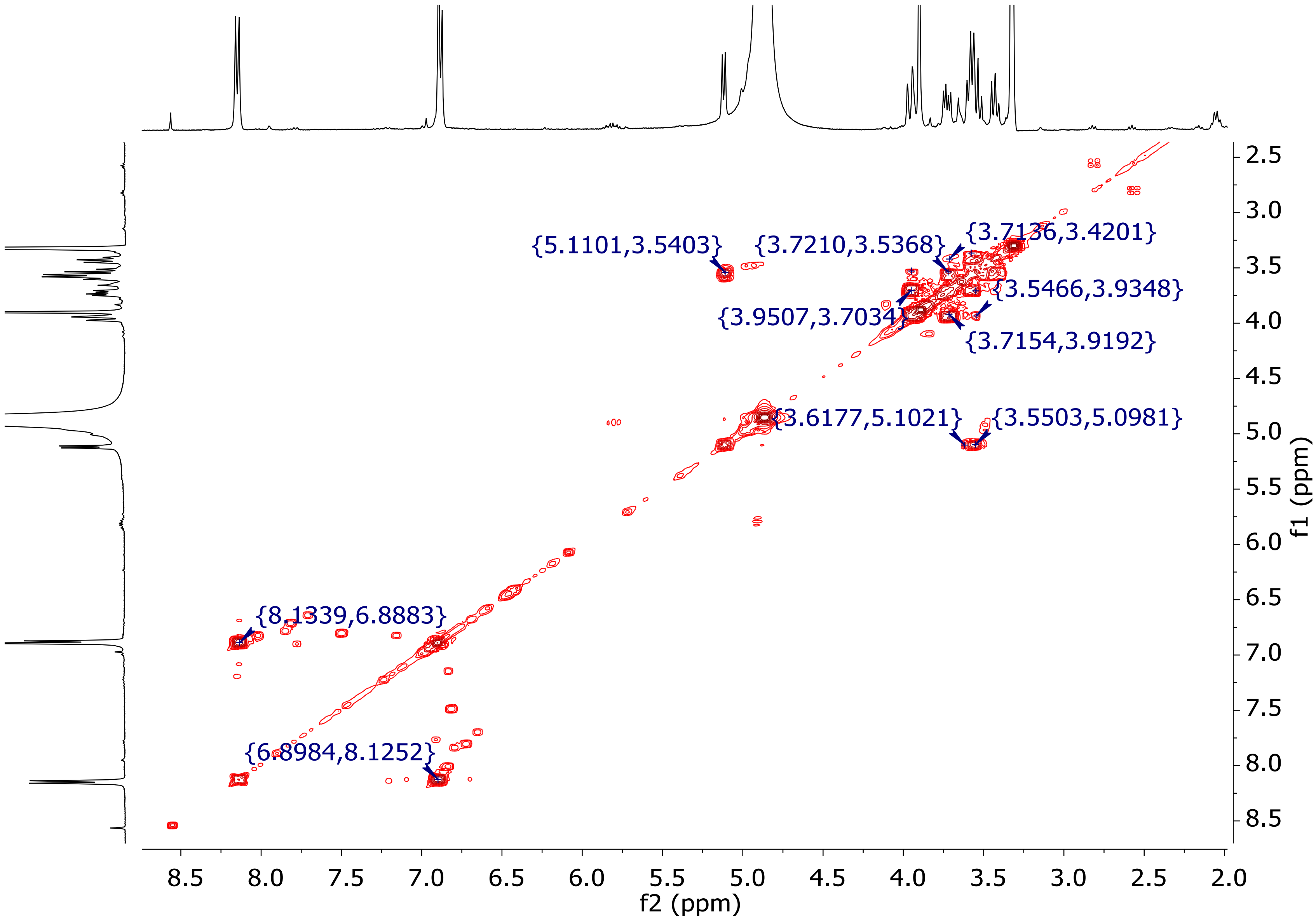

**Figure S30**. $^{1}H$ x $^{13}C$ (HSQC) NMR spectrum of compound **6**.

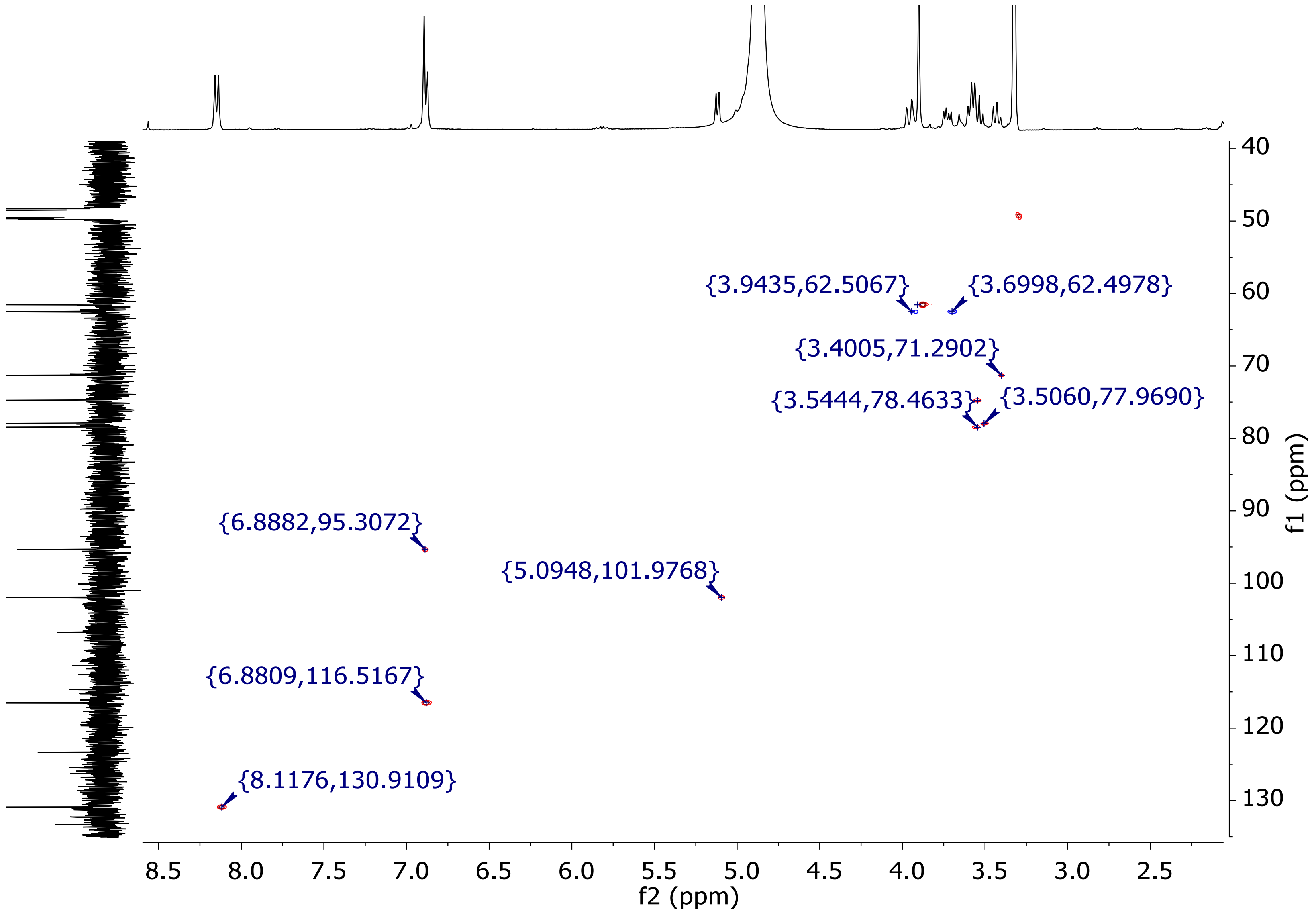

**Figure S31**. $^{1}H$ x $^{13}C$ (HMBC) NMR spectrum of compound **6**.

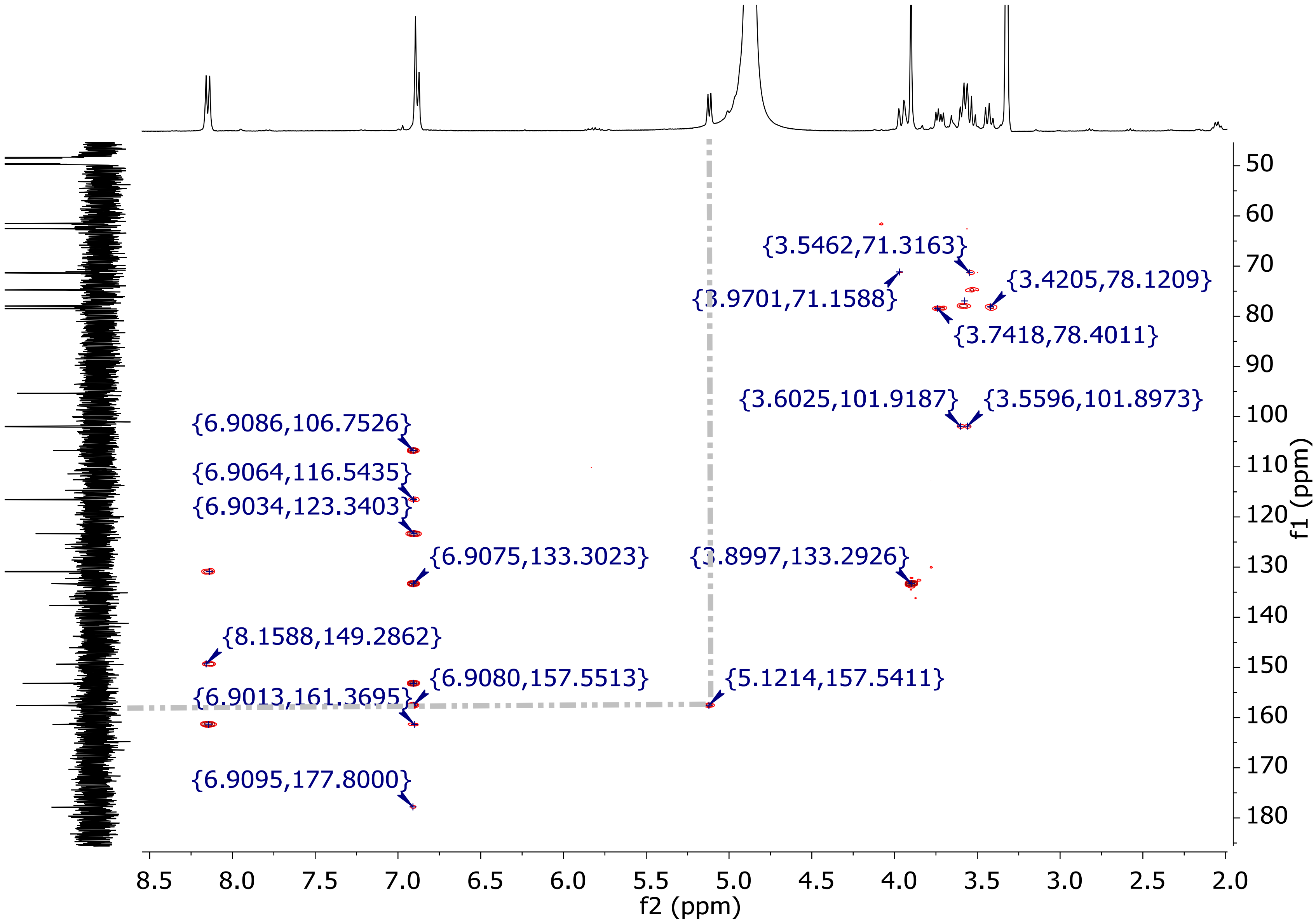

Paepalantine **(PbD-01)**: $^{1}$H NMR (400 MHz, $CD_3OD$) δ 2.29 (*s*, 3H, H-11), 3.86 (*s*, 3H, 7-$OCH_3$), 3.94 (*s*, 3H, 5-$OCH_3$), 6.52 (*s*, 1H, H-4), 6.56 (*s*, 1H, H-8), 6.90 (*s*, 1H, H-6), 9.53 (*sl*, 1H, 9-OH), 13.38 (*sl*, 1H, 10-OH).

**Figure S32**. $^{1}$H NMR spectrum (400 MHz, $CD_3OD$) of compound **PbD-01.**

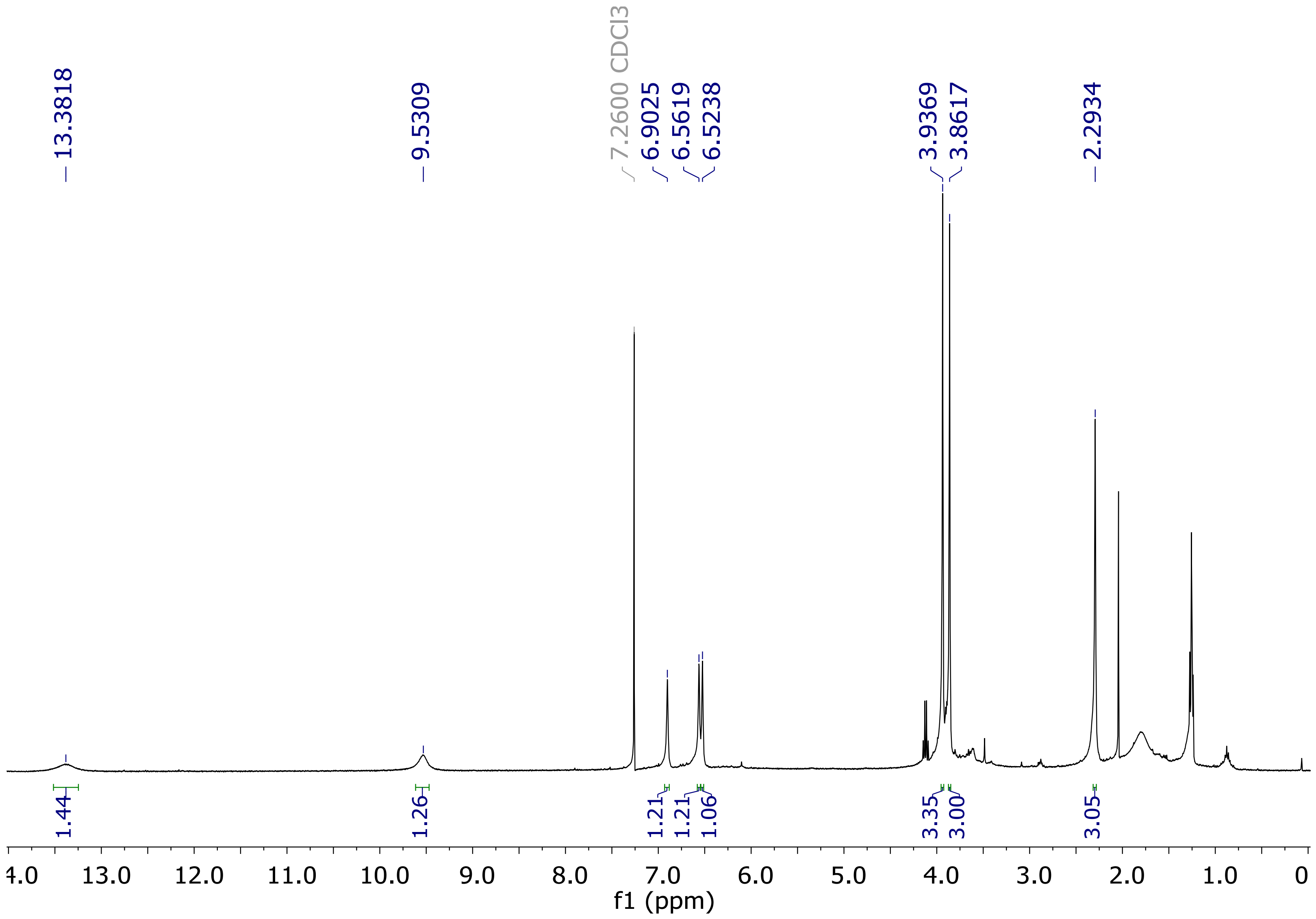

Supplement: Supplementary file 1 [file ao4c11026_si_001.pdf]
